# Pan-cancer analysis of co-inhibitory molecules revealing their potential prognostic and clinical values in immunotherapy

**DOI:** 10.3389/fimmu.2025.1544104

**Published:** 2025-03-24

**Authors:** Xiaoyu Ren, Anjie Guo, Jiahui Geng, Yuling Chen, Xue Wang, Lian Zhou, Lei Shi

**Affiliations:** ^1^ School of Life Sciences, Chongqing University, Chongqing, China; ^2^ Department of Head&Neck Cancer Center, Chongqing University Cancer Hospital, Chongqing, China

**Keywords:** immunotherapy, immune checkpoint inhibitors, co-inhibitory molecules, pan-cancer, biomarkers

## Abstract

**Background:**

The widespread use of immune checkpoint inhibitors (anti-CTLA4 or PD-1) has opened a new chapter in tumor immunotherapy by providing long-term remission for patients. Unfortunately, however, these agents are not universally available and only a minority of patients respond to them. Therefore, there is an urgent need to develop novel therapeutic strategies targeting other co-inhibitory molecules. However, comprehensive information on the expression and prognostic value of co-inhibitory molecules, including co-inhibitory receptors and their ligands, in different cancers is not yet available.

**Methods:**

We investigated the expression, correlation, and prognostic value of co-inhibitory molecules in different cancer types based on TCGA, UCSC Xena, TIMER, CellMiner datasets. We also examined the associations between the expression of these molecules and the extent of immune cell infiltration. Besides, we conducted a more in-depth study of VISTA.

**Result:**

The results of differential expression analysis, correlation analysis, and drug sensitivity analysis suggest that CTLA4, PD-1, TIGIT, LAG3, TIM3, NRP1, VISTA, CD80, CD86, PD-L1, PD-L2, PVR, PVRL2, FGL1, LGALS9, HMGB1, SEMA4A, and VEGFA are associated with tumor prognosis and immune cell infiltration. Therefore, we believe that they are hopefully to serve as prognostic biomarkers for certain cancers. In addition, our analysis indicates that VISTA plays a complex role and its expression is related to TMB, MSI, cancer cell stemness, DNA/RNA methylation, and drug sensitivity.

**Conclusions:**

These co-inhibitory molecules have the potential to serve as prognostic biomarkers and therapeutic targets for a broad spectrum of cancers, given their strong associations with key clinical metrics. Furthermore, the analysis results indicate that VISTA may represent a promising target for cancer therapy.

## Introduction

Cancer is a serious disease worldwide, and the inefficiency of existing therapies such as surgical cutting, radiotherapy, and chemotherapy is a hard nut to crack (1). Fortunately, cancer treatments are no longer restricted to conventional therapies since the loss of immune control has been proven to be a novel hallmark of cancer (2). Co-inhibitory receptors (IRs, also known as Immune Checkpoints), including CTLA4, PD-1, TIGIT, LAG3, TIM3, NRP1, VISTA, are crucial for regulating the duration and extent of immune response, thus in turn helping tumor cells to evade the surveillance of immune cells (3). Therefore, blocking these IRs as well as their ligands (here collectively referred to as co-inhibitory molecules) has emerged as a promising treatment option for numbers of human cancers.

Cytotoxic T lymphocyte-associated antigen-4 (CTLA4) is the first identified co-inhibitory molecules with high sequence similarity to CD28 (4). CD80 and CD86 are two of their ligands (5). The co-stimulation of CD28 by CD80/CD86 is essential for the transformation of resting T cells into effector T cells. Following the activation of the T cell receptor and CD28, intracellular CTLA-4 molecules translocate to the cell surface, where they competitively bind to CD80/86 with CD28. At the same time, CTLA-4 activates inhibitory signaling pathways and removes CD80/CD86 ligands through trans-endocytosis, leading to the suppression of T cell proliferation and activation (6–10). Programmed death receptor-1 (PD-1) is another marker of T-cell exhaustion (11) expressed on all activated T lymphocytes, B cells, monocytes, and dendritic cells (DCs) (12, 13). Programmed cell death ligand 1 (PD-L1) and 2 (PD-L2) have been reported to be ligands for PD-1. PD-1 primarily exerts its effects through Src homology 2 domain-containing phosphatase-2 (SHP2). When PD-1 binds to PD-L1/PD-L2, not only cytokine production but also T-cell differentiation will be depressed. In the same time, SHP2 can mediate the dephosphorylation of CD28 to inhibit T cell function (14). Furthermore, the coupling between PD-1 and PD-L1 impedes the interaction of T cells with DCs (15). Both PD-1 and CTLA4 can suppress T-cell response by downregulating Akt activity. The difference is that the CTLA4 pathway achieves this by involving the function of PP2A, while the PD-1 pathway does so by blocking PI3K activation (16). T cell Ig and ITIM domain (TIGIT) is a transmembrane protein receptor expressed on Natural Killer (NK) cells, CD8^+^ T cells and regulatory T (Treg) cells (17, 18). CD155 (PVR) and CD112 (PVRL2), currently known two ligands for TIGIT, are extensively expressed on tumor cells (19, 20). The other two receptors, CD226 and CD96, interact with the same ligands. Together with TIGIT, the three constitute complex signaling web where CD226 transmits a stimulatory signal (21), while CD96 and TIGIT deliver inhibitory signals (22). TIGIT not only competes for the ligand of CD226, but also binds it directly, thus disrupting its homodimerization and co-stimulatory function (17). Lymphocyte-activation gene 3 (LAG3), which is expressed on activated T cells and NK cells, negatively regulates the activation, proliferation of Th1 cells and its cytokine secretion (23). In addition to Major Histocompatibility Complex (MHC) Class II (24, 25), Galectin-3 (26), LSECtin (27), which have been reported to interact with LAG3, fibrinogen-like protein 1 (FGL1) is also a high-affinity ligand for it (28). The ligation of FGL1 to LAG3 decreases the levels of tumor necrosis factor alpha (TNF-α) and interferon (IFN)-β in plasma (28). Although the interaction modality of these ligands to LAG3 remains unclear, several studies have suggested that their binding contributes to the co-localization of LAG3 with the immune synapse, which is essential for its cytotoxic functions (29, 30). T cell immunoglobulin and mucin-domain containing-3 (TIM3) was originally found on CD4^+^ Th1 cells and CD8^+^ Tc1 (cytotoxic) cells, subsequently, TIM-3 has also been found on cells such as monocytes, natural killer (NK) cells, and dendritic cells. It is a part of the inhibitory receptor module (31, 32). In current research, four main ligands for TIM-3 have been identified: Galectin-9, high mobility group box 1 (HMGB1), Phosphatidylserine (PtdSer) and CEACAM1. The binding of LGALS9 to TIM3 has been shown to ensure the termination of effector Th1 (33) and induce CD8^+^ T cell apoptosis in colon cancer (34); On the other hand, the ligation of HMGB1 to TIM3 can suppress the activation of innate immune response by interfering with the binding of HMGB1 to receptor for advanced glycation end products and Toll-like receptors (35, 36); PtdSer is typically exposed on the surface of apoptotic cells. When it binds to TIM-3, it mediates the phagocytosis of apoptotic cells by macrophages, dendritic cells, and fibroblasts. However, in T cells, it only forms a complex with apoptotic material. Recent studies have shown that the binding of PtdSer to TIM-3 can stimulate the TIM-3 signaling pathway (32, 37); CEACAM1 induces immune tolerance by forming a dimer with TIM-3, which leads to the depletion of CD8+ T cells (38). Neuropilin-1 (NRP1), a multifunctional gene involved in both neural and vascular development (39), as well as in immunity and tumorigenesis, can not only maintain the stability of Treg cells (40) but also inhibit the anti-tumor function of CD8^+^ T cells (41). The obligation of SEMA4A to NRP1 enhances the stability of Treg cells by refraining the phosphorylation of Akt and boosting the nuclear localization of the transcription factor Foxo3a (40). Vascular endothelial growth factor (VEGF) is an immunosuppressive cytokine whose integration with NRP1 inhibits the maturation of DCs, which is essential to the efficiency of T-cell responses (42). VEGFA is a member of the VEGF superfamily, and the simultaneous blockade of VEGFA and NRP1 has been shown to have potent anti-tumor activity (43). V-domain Ig-containing suppressor of T cell activation (VISTA) is a recently identified immunomodulatory molecule homologous to PD-L1 (44, 45). It is expressed mainly on the cell surface of hematopoietic cells, myeloid cells, naïve CD4+ T cells and Foxp3(+)CD4(+) Treg cells (45–47). Notably, VISTA has been widely recognized as an inhibitory receptor not only for T cells but also for myeloid antigen-presenting cells and tumor cells (45), acting by reducing IFN-γ and TNF-α, restraining T-cell proliferation, inducing Foxp3 expression, and promoting the conversion of naïve T cells to Treg cells (48, 49). P-selectin glycoprotein ligand-1 (PSGL-1) and V-set and immunoglobulin domain containing 3 (VSIG3) are the most studied ligands of VISTA. Under physiological pH, VISTA interacts with VSIG3, inhibiting T cell function and reducing immune cell infiltration; Under acidic pH conditions, VISTA binds to PSGL-1 on T cells, which is associated with immune tolerance in the acidic tumor microenvironment (50–53). Previous studies have suggested that VISTA plays a crucial role in the homeostasis of naïve T cells and contributes to immune tolerance. When VISTA is deficient, it disrupts T cell quiescence and enhances immune responses (54). Recent studies have shown that VISTA, expressed in the tumor microenvironment (on antigen-presenting cells or tumor cells), is also involved in immune evasion. It interacts with LRIG1, which is expressed on activated tumor-specific CD8+ T cells, either in cis (within the same cell) or in trans (between different cells), activating inhibitory signals that lead to a reduction in CD8+ T cells and their entry into a quiescent state (55). Nevertheless, the unclear role of VISTA on the cell surface adds to its complexity and contributes to the controversy surrounding its function.

Already in 1996, Leach et al. proposed that the blockade of co-inhibitory molecules could be an advanced strategy of cancer treatment (56). The first commercialized immune checkpoint inhibitors (ICIs), Ipilimumab, an anti-CTLA4 monoclonal antibody (mAb), was approved by the US Food and Drug Administration (FDA) in 2011 for the treatment of melanoma, marking a crucial step in cancer immunotherapy (57–59). ICIs, including anti-CTLA4/PD-1/PD-L1 mAbs, have evolved over the past few years as anti-cancer treatment options and have become one of the most successful cancer therapies (60). Nonetheless, these agents do not work in all patients (61, 62). Great hopes therefore have been placed onto additional targets (63). Meanwhile, the development of combination therapeutic strategies is also vital for tumor therapy, and some have already demonstrated efficiency in partial cancer patients (64–66). For instance, treatment with Ipilimumab plus Nivolumab (anti PD-1) has shown durable survival benefits in various tumor types, such as Small-Cell Lung Cancer (SCLC) (67), malignant pleural mesothelioma (MPM) (68), advanced melanoma (69), Hepatocellular Carcinoma (HCC) (70), etc. Therefore, in order to facilitate the development of novel therapeutic approaches and individualized therapies, it would be helpful to perform a pan-cancer analysis of the co-inhibitory molecules that could clearly and efficiently reveal the significance of specific genes in various cancer types (71). However, there are relatively few studies of this type. To this end, in this study, we investigated the expression, correlation, and prognostic value of co-inhibitory molecules in different cancer types based on TCGA, UCSC Xena, TIMER, CellMiner datasets. We also examined the associations between the expression of these molecules and the extent of immune cell infiltration. Building on these studies, we specifically focused on VISTA. These results might provide important insights into the role of co-inhibitory molecules in antitumor immunity.

## Materials and methods

### Data download

We used the Cancer Genome Atlas (TCGA, https://www.cancer.gov/tcga.), an online dataset collecting over 20,000 samples from 33 primary cancers and matched normal samples, to download the pan-cancer (Adrenocortical carcinoma, ACC; Bladder urothelial carcinoma, BLCA; Breast invasive carcinoma, BRCA; Cervical squamous cell carcinoma and endocervical adenocarcinoma, CESC; Cholangiocarcinoma, CHOL; Colon adenocarcinoma, COAD; Lymphoid Neoplasm Diffuse Large B-cell Lymphoma, DLBC; Esophageal carcinoma, ECSA; Glioblastoma multiforme, GBM; Head and Neck squamous cell carcinoma, HNSC; Kidney Chromophobe, KICH; Kidney renal clear cell carcinoma, KIRC; Kidney renal papillary cell carcinoma, KIRP; Acute Myeloid Leukemia, LAML; Brain Lower Grade Glioma, LGG; Liver hepatocellular carcinoma, LIHC; Lung adenocarcinoma, LUAD; Lung squamous cell carcinoma, LUSC; Mesothelioma, MESO; Ovarian serous cystadenocarcinoma, OV; Prostate adenocarcinoma, PAAD; Pheochromocytoma and Paraganglioma, PCPG; Prostate adenocarcinoma, PRAD; Rectum adenocarcinoma, READ; Sarcoma, SARC; Skin Cutaneous Melanoma, SKCM; Stomach adenocarcinoma, STAD; Testicular Germ Cell Tumors, TCTG; Thyroid carcinoma, THCA; Thymoma, THYM; Uterine Corpus Endometrial Carcinoma, UCEC; Uterine Carcinosarcoma, UCS; and Uveal Melanoma, UVM) transcription expression data, survival data, mutation data, stemness score, and DNA/RNA methylation regulatory genes expression data (72) (71). We then downloaded the immune infiltration estimations of pan-cancer from Tumor Immune Estimation Resource (TIMER, http://timer.cistrome.org/), which applies a deconvolution statistical approach to evaluate immune cell infiltration in different types of cancer based on gene expression data (73, 74). CellMiner database (https://discover.nci.nih.gov/cellminer/home.do) (75), screening over 100,000 chemical compounds and natural products, was used here to obtain relevant information about drug sensitivity.

### Differential expression analysis

To investigate the expression levels of co-inhibitory molecules in different cancers and corresponding normal tissues, the TCGA and TIMER databases were used. We employed “DEseq2” R package to calculate the fold-changes (76), which can be used to represent multiples of the differential expression of the genes, and P-value generated from the hypothesis test was corrected with the Benjamini-Hochberg algorithm to obtain the False Discovery Rate (FDR) (77). Here we set FDR <= 0.05 as an indicator of statistical significance. Furthermore, the values of ​Transcripts Per Million (TPM) for co-inhibitory molecules, which compare the proportion of reads mapped to the genes in each sample and were displayed in the form of boxplots, were downloaded from TIMER.

### Correlation analysis of co-inhibitory molecules

The next correlation analysis was conducted to identify the correlation between co-inhibitory molecules. First, based on the expression data downloaded from TCGA, we used the “Hmisc” R package, the Pearson method to calculate the correlation coefficients between each two genes in all tumor samples (78), and the “ggcorrplot” package to visualize the correlation results. Additionally, we performed the pairwise correlation analysis of co-inhibitory molecules in individual tumor types by using the “cor.test” function and “ggplot2” R package. It is generally considered that the absolute value of the correlation coefficient above 0.8 means a strong correlation, between 0.3 and 0.8 means a weak correlation, and below 0.3 refers to no correlation.

### Survival analysis

Survival analysis was based on TCGA and UCSC Xena browser (https://xenabrowser.net/). Major indicators assessed in our research included Overall Survival (OS), Progression-Free Interval (PFI), and Disease-Specific Survival (DSS) (79). We used a univariate Cox proportional hazards model (“Survival” R package) to calculate the hazard ratio (HR) between the expression levels of co-inhibitory molecules and patients OS as well as PFI, where P <= 0.05 served as a cut-off for statistically significance (80). The Kaplan-Meier curves of the OS, PFI, and DSS were analyzed based on web tools and downloaded from UCSC Xena, a high-performance analysis and visualization tool (81).

### Relationship between co-inhibitory molecules and immune cell infiltration

Information on the infiltration of immune cells (B cell, CD4^+^ T cell, CD8^+^ T cell, Neutrophil, Macrophage, Myeloid dendritic cell) in each tumor type under the six algorithms (TIMER (82), CIBERSORT (83), QUANTISEQ (84), MCP-COUNTER (85), XCELL (86), EPIC (87)) was obtained from TIMER database. We used Spearman’s approach (88) to investigate the correlations of co-inhibitory molecules and the infiltration of different types of immune cells in pan-cancer under the TIMER algorithm, then further compare them under six algorithms, where P <= 0.05 was considered statistically significant.

### VISTA expression and TMB, MSI, Stemness in pan-cancer

Tumor Mutation Burden (TMB) referring to the number of mutations in a specific cancer genome and Microsatellite Instability (MSI) measuring the frequency of the simple sequence repeat (SSR) length variation can serve as indicators of patients’ response to ICIs (89–93). Besides, Cancer cell stemness, which measures the levels of the stem cell transcriptome (mRNAsi) and the methylome (mDNAsi), is regarded as a biomarker of negative survival outcome (94, 95). Hence, we directly obtained tumor mutation and cancer cell stemness data from TCGA and MSI values from analysis conducted by Russell et al. (96), and then used Spearman’s method to analyze the correlation between VISTA and the three indexes. Both metrics were visualized using a radar map designed by the R-package “ggradar”.

### Correlation of VISTA and methylation regulatory genes

Methylation of DNA and RNA occurs mainly in the forms of 5-methylcytosine (5mC) and N6-methyladenosine (m6A) respectively (97, 98). DNA methylation is catalyzed by DNA methyltransferases (DNMTs), including DNMT3 and DNMT1 families which are responsible for methylation establishment and maintenance (99, 100). Human AlkB homolog H5 (ALKBH5) (101), Fat Mass and Obesity-associated protein (FTO) (102) are primary m6A demethylases. Methyltransferase like 3 (METTL3), METTL14, and Wilms tumor 1-associated protein (WTAP) contribute to the m6A modification process initiation (103). We downloaded the expression values of DNA/RNA methylation genes in pan-cancer from the TCGA database, analyzed their correlation coefficients with co-inhibitory molecules using the Pearson method, “Hmisc” package, and applied the R package “ggplot2” to plot circular graphs for visualization.

### Drug sensitivity analysis and clinical trials on VISTA

For drug sensitivity analysis, the expression values of co-inhibitory molecules as well as the drug sensitivity data were obtained from the CellMiner database (104, 105). Next, we used “ggstatsplot” R package to calculate their correlation coefficients based on the Pearson and Hedges methods, and then visualized them through scatter and violin plots respectively.

### Clinical trials

Information on clinical trials regarding VISTA was collected from ClinicalTrials.gov (https://clinicaltrials.gov), which explores 408,952 research studies in 220 countries by the time of our study and assists investigators and patients to understand the currently available research trials.

## Results

### Expression of the co-inhibitory molecules across different cancers

**Figure 1:**
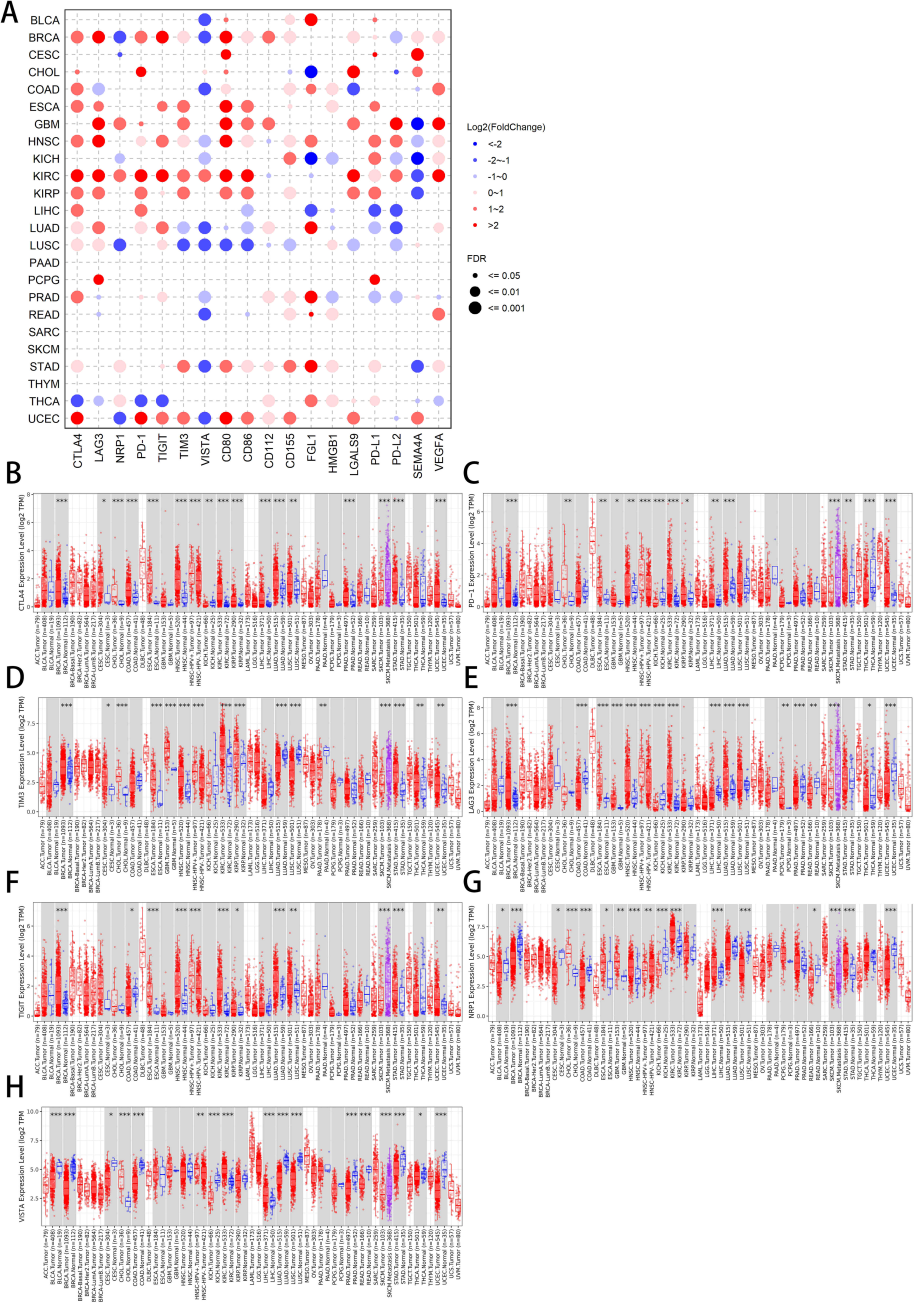
Differential expression analysis of co-inhibitory molecules across TCGA pan-cancers. **(A)** Log2(FoldChange) of 18 co-inhibitory molecules in 24 available TCGA cancers. The red and blue bubbles represent their high or low expression in indicated cancers, respectively. The size of the points indicates the value of False Discovery Rate (FDR), and data with FDR greater than 0.05 are not displayed. **(B–H)** The expression of 7 co-inhibitory receptors [CTLA4 **(B)**, PD-1 **(C)**, TIM3 **(D)**, LAG3 **(E)**, TIGIT **(F)**, NRP **(G)**, and VISTA **(H)**] in different cancer types and corresponding normal tissues. Blue boxes represent normal tissues while red one represent cancer tissues, and each dot indicates one sample. * p < 0.05, ** p < 0.01, *** p < 0.001.

The co-inhibitory molecules play an important role in the tumor microenvironment (TME) by contributing to T cell exhaustion and immune escape. It could be hypothesized that these molecules might have unique expression landscapes in various immune cells, in order to perform their respective functions. The specific expression of these molecules in immune cells involving Granulocytes, Monocytes, T cells, B cells, DCs, NK cells, and Progenitors was shown in the **Supplementary Figure 1A**. CTLA4, LAG3, PD-1, TIGIT were mainly expressed on T cells, whereas CD80 was highly expressed on B cells, and VISTA as well as CD155 were primarily expressed on Granulocytes and Monocytes. Noticeably, the expression of HMGB1 in the above immune cells was relatively even and high. These results suggested that the expression of co-inhibitory molecules was not limited in effector cells, so their roles in anti-tumor immunity needed to be further investigated. We then compared the mRNA levels of these molecules in tumor and normal tissues. **Figure 1A** illustrates the landscape of the log2(fold change) (FC) of these 18 genes across 24 TCGA cancers with available tumor and normal tissues. CTLA4, LAG3, PD-1, TIGIT, TIM3, CD80, CD86, CD112, CD155, FGL1, PD-L1 and VEGFA tended to have log2 (FC) values greater than zero, while the values of log2 (FC) of VISTA are mostly below zero (p <= 0.05).

Then, we searched the TIMER platform, providing the visualization of pan-cancer expression profiling of co-inhibitory molecules. CTLA4 and PD-1 expression levels were upregulated in tumor tissues relative to non-carcinoma tissues in BRCA, CHOL, ESCA, HNSC, KIRP, LIHC, LUAD, STAD, UCEC, whereas both were downregulated in KICH. PD-1 was also upregulated in KIRC and downregulated in THCA (**Figures 1B, C**). This almost identical expression pattern demonstrates that CTLA4 and PD-1 may be strongly correlated in different types of tumors, indicating a possible similar role in anti-tumor immunity. The expression of TIM3 in BRCA, CESC, CHOL, ESCA, GBM, HNSC, KIRC, KIRP, STAD, THCA and UCEC tumor cells was significantly higher than that in normal cells, while its expression levels in LUAD and LUSC tumor cells were lower than normal cells (**Figure 1D**). The specific expression of TIM3 in lung cancer may suggest differences when it plays its role. LAG3 and TIGIT expression levels were upregulated in tumor tissues in BRCA, ESCA, HNSC, KIRC, LUAD, LUSC, while both were downregulated in COAD and SKCM. LAG3 was also upregulated in GBM, PCPG and downregulated in KICH, LIHC, PRAD, READ, THCA, UCEC, whereas TIGIT was additionally upregulated in KIRP, STAD, UCS (**Figures 1E, F**). Not only did the two show relatively high similarity in differential expression, but also in prognostic studies. The expression of NRP1 in CHOL, ESCA, GBM, HNSC, KIRC, LIHC, STAD tumor cells was significantly higher than that in normal cells, while its expression levels in BLCA, BRCA, CESC, COAD, KICH, LUSC, READ, SKCM, UCEC tumor cells were lower (**Figure 1G**). The expression of VISTA in CHOL, KIRC and LIHC tumor cells was higher than normal cells. In BLCA, BRCA, CESC, COAD, KICH, LUAD, LUSC, PRAD, READ, STAD and UCEC tumor cells, interestingly, the opposite phenomenon was true (**Figure 1H**). From the expression perspective alone, VISTA had already shown great differences from other co-inhibitory molecules. More detailed information about the ligands was available in the **Supplementary Figure 1B** and **Supplementary Table 1**.

### Correlation analysis of co-inhibitory molecules

**Figure 2:**
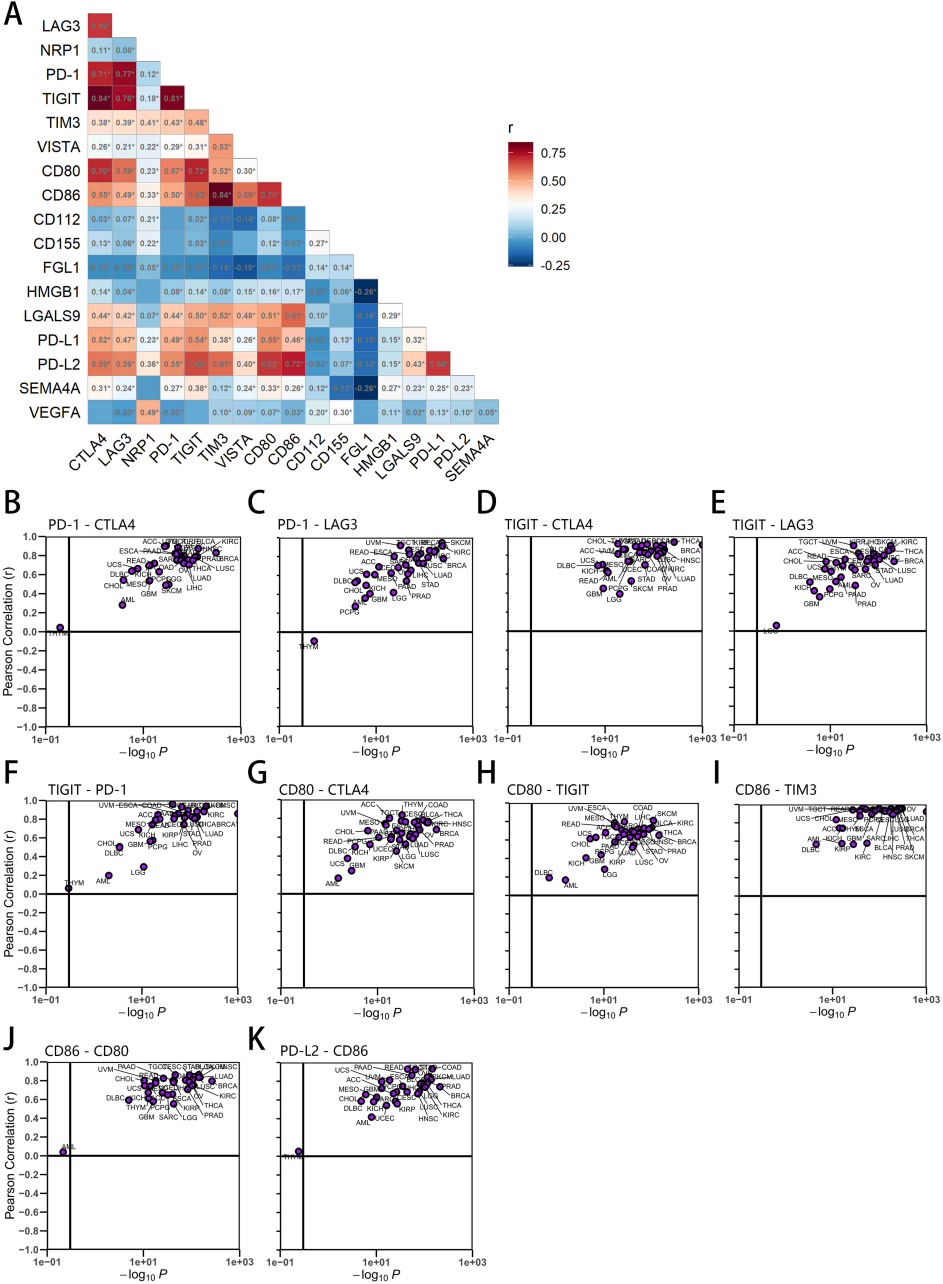
Heatmap of correlation between co-inhibitory molecules in all samples and individual tumor types. **(A)** Red or blue boxes indicates a positive or negative correlation coefficient between two molecules in all samples. The shades of color in the graph indicate the level of coefficient, while the significant values are marked in their respective squares. **(B-K)** The top 10 combinations with the highest correlation coefficient were further analyzed by tumor type and were consistent with each other except for certain individual tumor types. **(B)** The correlation coefficient between PD-1 and CTLA4 based on all samples was 0.71 (* p < 0.05, as per **A**, same below), while the coefficient by tumor type was between 0.3 and 1.0 except for THYM. **(C)** PD-1 and LAG3 (All samples: r=0.77, Tumor types: r:0.3-1 except for THYM). **(D)** TIGIT and CTLA4 (All samples: r=0.84, Tumor types: r: 0.4-1). **(E)** TIGIT and LAG3 (All samples: r=0.76, Tumor types: r: 0.3-1 except for LGG). **(F)** TIGIT and PD-1 (All samples: r=0.81, Tumor types: r: 0.4-1 except for LGG, AML, THYM). **(G)** CD80 and CTLA4 (All samples: r=0.70, Tumor types: r: 0.3-1 except for GBM, AML). **(H)** CD80 and TIGIT (All samples: r=0.72, Tumor types: r: 0.3-1 except for DLBC, AML). **(I)**CD86 and TIM3 (All samples: r=0.84, Tumor types: r: 0.4-1). **(J)** CD86 and CD80 (All samples: r=0.70, Tumor types: r: 0.4-1 except for AML). **(K)** PD-L2 and CD86 (All samples: r=0.72, Tumor types: r: 0.4-1 except for THYM).

As suggested by the above analysis, there is a relationship between the expression of co-inhibitory genes, such as CTLA4 and PD-1, in different types of tumors, which prompted us to think about the correlation between them, so we next performed a correlation analysis between these genes. In fact, co-inhibitory molecules are not insulated from each other. Evidence has suggested that the blockade of one co-inhibitory molecules probably alter the sensitivity of another to its antibody, thus influencing the effect of treatment (17, 106–108). Hence, these published data also indicate that it is meaningful to study the correlation between co-inhibitory molecules. As we can see from **Figure 2A**, CTLA4 was significantly correlated with TIGIT (R = 0.84), PD-1 (R = 0.71), CD80 (R = 0.70), and LAG3 (R = 0.69). PD-1 was significantly correlated with TIGIT (R = 0.81), and LAG3 (R = 0.77). TIGIT had a clear correlation with LAG3 (R = 0.76), CD80 (R = 0.72), and PD-L2 (R = 0.66). TIM3 was clearly correlated with CD86 (R = 0.84), and PD-L2 (R = 0.61). CD80 was obviously correlated with CD86 (R = 0.70), and PD-L2 (R = 0.68). CD86 had a dramatically positive correlation with PD-L2 (R = 0.72), LGALS9 (R = 0.61). PD-L1 was clearly correlated with PD-L2 (R = 0.69) (all p < 0.05). These correlations are consistent with the results of the previously mentioned analyses and the available literature. However, the above analysis is based on samples from the whole TCGA data, and according to the literature, sometimes different types of tumors may exhibit different expression profile characteristics. To further validate the correlation results, we further investigated the 10 combinations with the highest correlation coefficient to explore their correlations in individual tumor types. Overtly, they showed a high correlation in most cancer types (**Figures 2B–K**). Nonetheless, interestingly, these combinations of PD-1 and CTLA4 (**Figure 2B**), PD-1 and LAG3 (**Figure 2C**), PD-1 and TIGIT (**Figure 2F**), PD-L2 and CD86 (**Figure 2K**) all showed an exceptionally low correlation in THYM (close to or even below zero), with the correlation of TIGIT and LAG3 as well as CD80 and CD86 showing a similar phenomenon in LGG and AML, respectively (**Figures 2E, J**). Notably, the combination of anti-LAG3 and anti-PD-1 was recently approved by the FDA for the treatment of patients with unresectable or metastatic melanoma, as also significantly noted in our analysis (**Figure 2C**) (109).

### Survival analysis of co-inhibitory molecules

**Figure 3:**
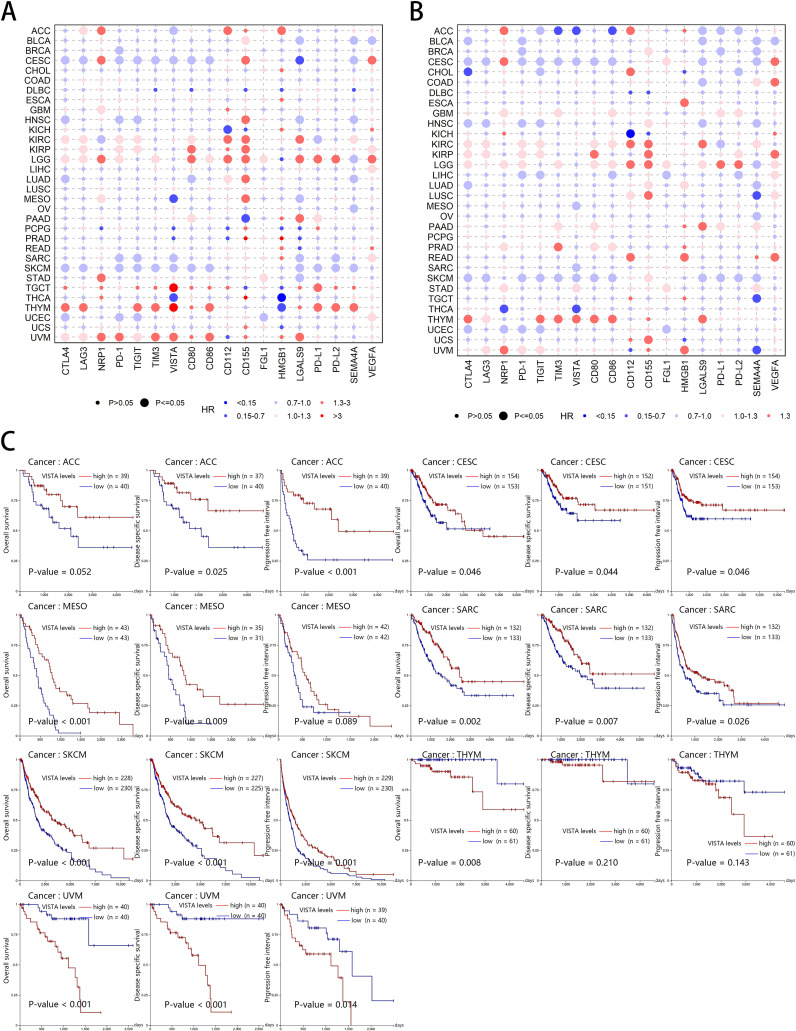
Survival analysis of co-inhibitory molecules across multiple cancer types. **(A)** The Bubble diagram of Hazard Ratio (HR) of 18 co-inhibitory molecules to overall survival (OS) in 32 TCGA cancer types. **(B)** The Bubble diagram of HR of 18 co-inhibitory molecules to Progression-Free Interval (PFI) in 32 TCGA cancer types. HR and P-value in the figures were calculated based on univariate Cox proportional hazards model. HR is indicated by color, where a darker red color indicates a larger value and the opposite in blue, and statistically significant P-value (<0.05) is indicated by smaller circles. **(C)** Association between VISTA expression and survival in ACC, CESC, MESO, SARC, SKCM, THYM, and UVM. Red and blue lines indicate high and low VISTA expression, respectively. The P-value (set P-value < 0.05 as the threshold value) is shown directly in the bottom left corner of each figure.

The relationship between the expression levels of some co-inhibitory molecules and the survival of tumor patients has been widely and intensively studied. However, not every gene has been studied in depth, and at the same time, there is a lack of systematic survival analysis for all co-inhibitory molecule genes. To better understand their prognostic value, we next investigated the relationships between co-inhibitory molecules expression and cancer prognosis. Based on COX analysis, we first explored the overall implications of OS (**Figure 3A**) and PFI (**Figure 3B**) using the data from TCGA. Although somewhat complicated, we can obtain prognostic indicators for each gene from this analysis and make comparative observations between them. CTLA4 was indicated as a prognostic factor both for OS and PFI in 8 cancers (5 positive *vs.* 3 negative), LAG3 (2 positive *vs.* 4 negative), NRP1 (0 positive *vs.* 6 negative), PD-1 (5 positive *vs.* 4 negative), TIGIT (4 positive *vs.* 4 negative), TIM3 (2 positive *vs.* 2 negative), VISTA (6 positive *vs.* 1 negative), CD80 (2 positive *vs.* 4 negative), CD86 (2 positive *vs.* 2 negative), CD112 (1 positive *vs.* 3 negative), CD155 (0 positive *vs.* 5 negative), FGL1 (1 positive *vs.* 2 negative), LGALS9 (4 positive *vs.* 3 negative), PD-L1 (2 positive *vs.* 2 negative), PD-L2 (1 positive *vs.* 1 negative), SEMA4A (7 positive *vs.* 1 negative), VEGFA (1 positive *vs.* 4 negative). Notably, the majority of co-inhibitory molecules is threatening to the prognosis of KIRC, KIRP, LGG, THYM, and UVM patient. All data can be found in **Supplementary Tables 2**, **3**.

The above results were obtained from downloaded data for local COX analyses. To verify the accuracy and to further validate these results, we performed KM analyses based on the widely recognized online tool Xena. Based on hazard ratio (HR) values above, we show the KM diagram with significant logRank P values, indicating that our COX and KM analysis are consistent. Taking VISTA as an example, we were unexpected to find that VISTA usually play a positive role in tumor cells, such as ACC (OS: P-value = 0.051; DSS: P-value = 0.025; PFI: P-value < 0.001), CESC (OS: P-value = 0.046; DSS: P-value = 0.044; PFI: P-value = 0.046), MESO (OS: P-value < 0.001; DSS: P-value = 0.009; PFI: P-value = 0.089), SARC (OS: P-value = 0.002; DSS: P-value = 0.007; PFI: P-value = 0.026), SKCM (OS: P-value < 0.001; DSS: P-value <0.001; PFI: P-value = 0.001), which is consistent with the literature (110), Nonetheless, elevated VISTA expression had negative impacts on survival in THYM (OS: P-value = 0.008; DSS: P-value = 0.210; PFI: P-value = 0.143), and UVM (OS: P-value < 0.001; DSS: P-value < 0.001; PFI: P-value = 0.014) (**Figure 3C**). The result suggested that the role of VISTA was heterogeneous in prognosis across different cancer types and it may not be suitable for a therapeutic target in some tumors.

### Relationship between co-inhibitory molecules and immune cell infiltration

**Figure 4:**
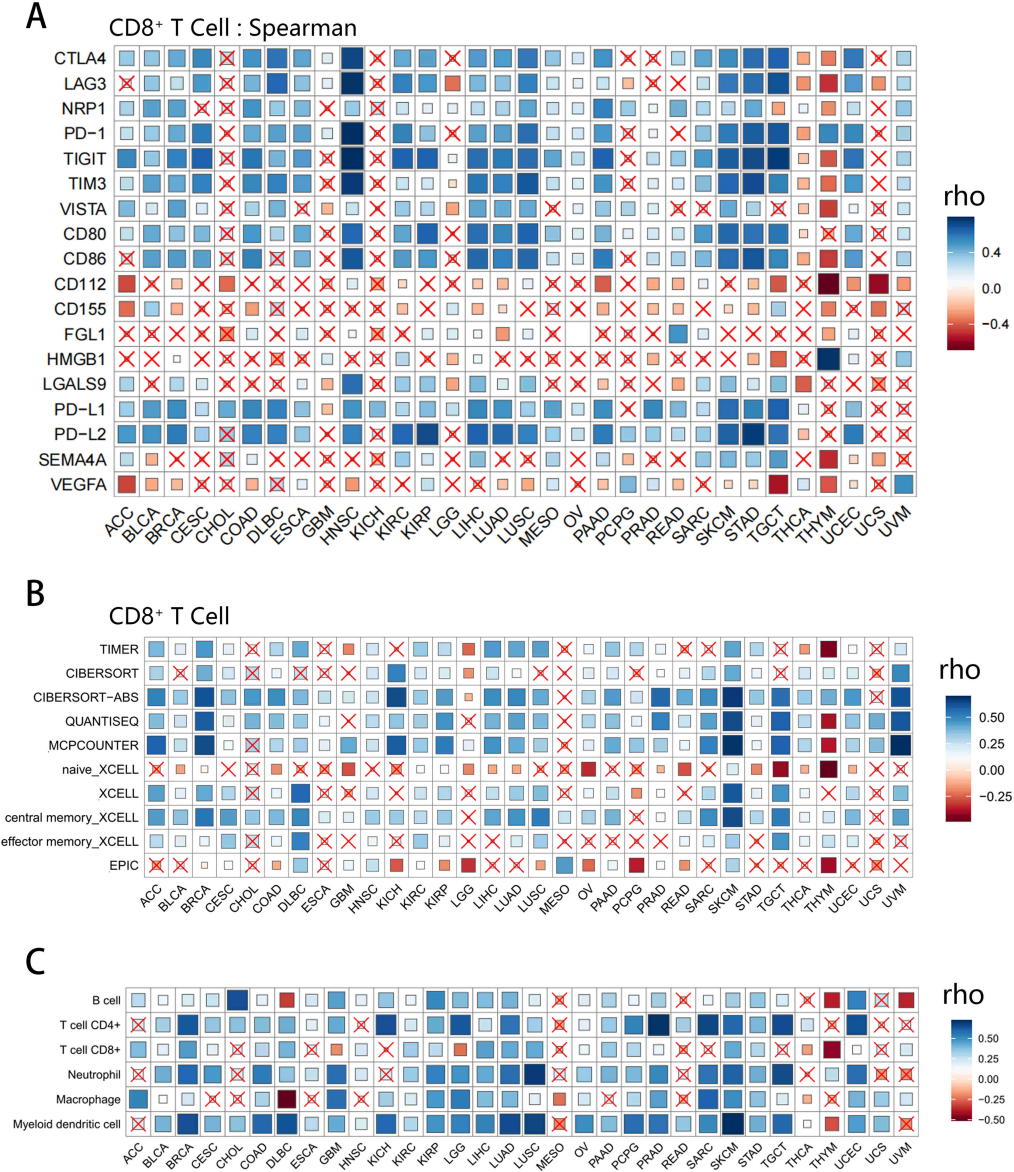
Correlation analysis of the expression of co-inhibitory molecules and pan-cancer immune cell infiltration. Data with P-values greater than the threshold (P > 0.05, not significant) were cross labeled. **(A)** The correlation between 18 molecules and CD8^+^ T cell infiltration in different tumor types based on Spearman method. Inhibitory receptors (CTLA4, LAG3, TIGIT, TIM3, PD-1, NRP1, VISTA) are positively correlated with CD8^+^ T cell infiltration in most tumors except for THCA and THYM. **(B)** The relationship between the VISTA expression and the CD8^+^ T cell infiltration levels under 6 different algorithms (TIMER, CIBERSORT, QUANTISEQ, MCPCOUNTER, XCELL, EPIC), where some differences can be seen due to the different algorithm and sample availability. **(C)** Correlation between VISTA expression and B cell, CD4^+^ T cell, CD8^+^ T cell, Neutrophil, Macrophage, Myeloid dendritic cell infiltration levels. Despite being different types of immune cells, the correlations of VISTA with them are highly consistent, highlighting the uniqueness of THYM.

The intensive research on immunotherapy in recent years has made us realize the importance of TME, especially the infiltration of immune cells. Among these, the CD8^+^ T cells are gaining increasingly popularity due to their anti-tumor effect and potential to function as a favorable prognostic biomarker in solid tumors (111–114). Overview of the relationship between co-inhibitory molecules and CD8^+^ T cells infiltration was shown in **Figure 4A** (As for other immune cells, the corresponding information can be seen in **Supplementary Figure 2**). As we can see in most cancer types, IRs are almost positively correlated with CD8^+^ T cell infiltration. On the other hand, in terms of ligands, PD-L1, PD-L2, CD80, CD86 and LGALS9 were positively associated with CD8^+^ T cell infiltration, whereas PVR, PVRL2, VEGFA generally had negative correlation with it. What caught our attention was that in THCA as well as THYM, the expression of co-inhibitory molecules was usually associated with poor CD8^+^ T cell infiltration. To further validate the above results, we used different algorithms, such as TIMER, Cibersort, QUANTISEQ and MCPCOUNTER, to assess T-cell infiltration and the analyses showed relatively high agreement between these algorithms (**Figure 4B**).

Thus, a new question was raised. Since the infiltration of CD8^+^ T cells is related to co-inhibitory molecules, how about the contribution of other immune cells? Although T cells are the most important immune infiltrating cells in tumor immunity, other cells, such as DCs, are also thought to be involved in the immune regulation and tumor clearance of TME. We moved on to investigate the association between these molecules and B cell, CD4^+^ T cell, Neutrophil, Macrophage, Myeloid dendritic cell infiltration (**Supplementary Tables 4** and **5** displayed the correlation of 18 co-inhibitory molecules with various immune cells from TIMER and CIBERSORT, respectively). Here, we presented the correlation plot between VISTA and six types of immune cells (**Figure 4C**). Overall, VISTA showed a positive correlation with immune cell infiltration in various cancers, while THYM was the obvious exception. Given all this, the role of VISTA is more elusive and worth further exploring.

### Correlation analysis of VISTA and TMB, MSI in pan-cancer

**Figure 5:**
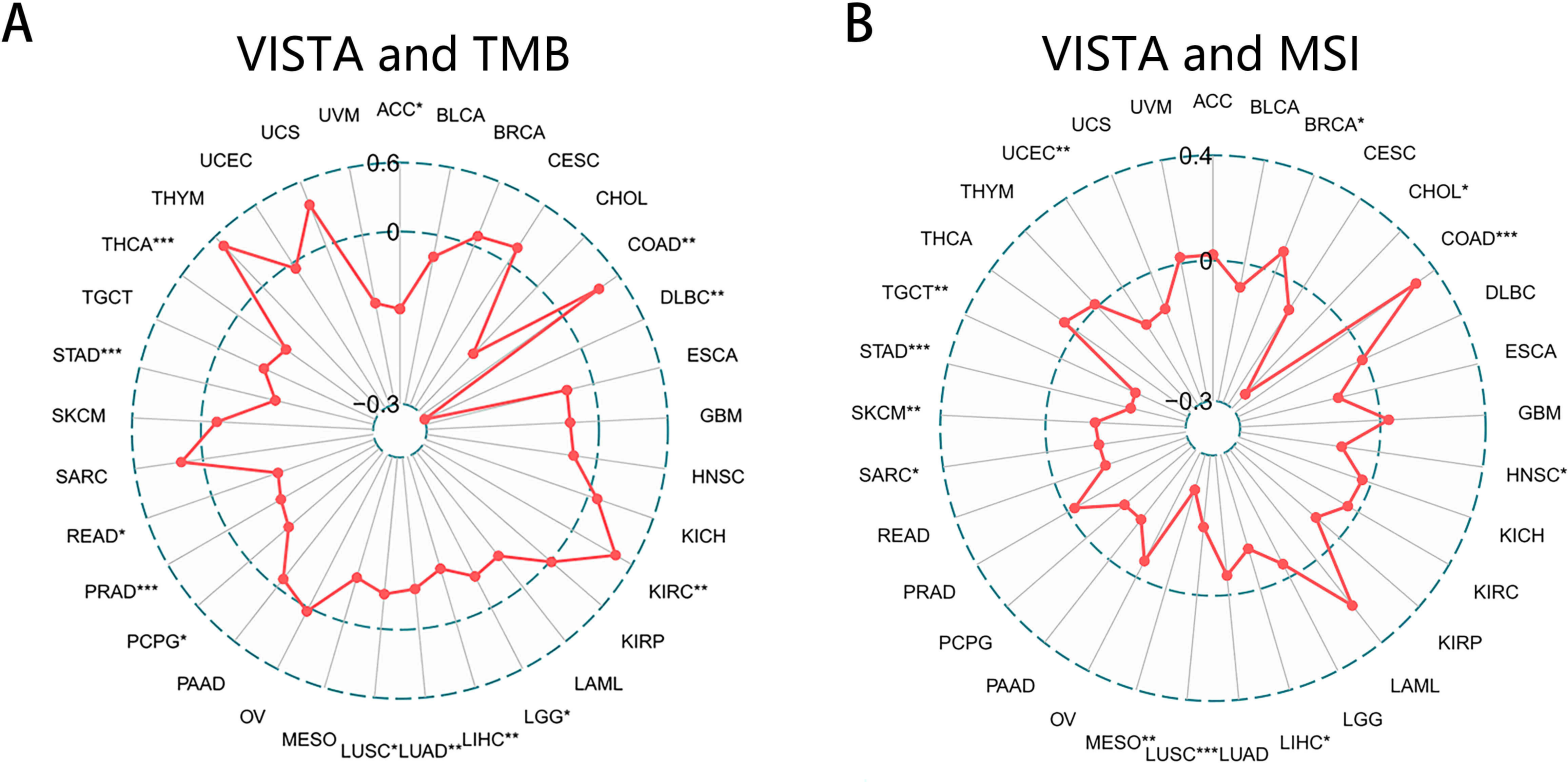
Correlation of VISTA expression with TMB and MSI. **(A)** TMB score for each sample was calculated based on genomic mutations. The radar chart exhibiting the correlation between VISTA and TMB in 33 cancers. **(B)** Relation between VISTA and MSI. MSI score was used to assess changes in simple sequence repeats, which can be detected by multiple fluorescence PCR and capillary electrophoresis. The points represented the Spearman correlation coefficient for each tumor type. * p < 0.05, ** p < 0.01, *** p < 0.001.

Although there is no comprehensive evidence that TMB or MSI is associated with every co-inhibitory molecule, they are both important factors in tumor immunotherapy (89–93) and can even serve as an emerging biomarker associated with the sensitivity to ICIs such as PD-1/PD-L1 mAbs (92, 115, 116). Hence, we were very interested in whether the expression of these genes is associated with TMB or MSI. Since VISTA was observed to have important effects on the tumor prognosis and TME, here we focused on the correlation between VISTA and TMB as well as MSI to further explore the role of VISTA in tumors (Relevant information about other genes was presented in the **Supplementary Table 6**). Our results indicated that the higher the expression of VISTA, the lower TMB in ACC (r = -0.225), DLBC (r = -0.499), LIHC (r = -0.160), PCPG (r = -0.151), PRAD (r = -0.179), READ (r = -0.203), STAD (r = -0.206), THCA (r = -0.172), while the higher TMB in COAD (r = 0.132), KIRC (r = 0.145) (**Figure 5A**). With respect to MSI, VISTA had a positive association with it in COAD (r = 0.233), whereas a negative correlation could be seen in CHOL (r = -0.344) LUSC (r = -0.195), MESO (r = -0.295) STAD (r = -0.236), TGCT (r = -0.235) (**Figure 5B**).

### Correlation analysis of VISTA and cancer cell stemness

**Figure 6:**
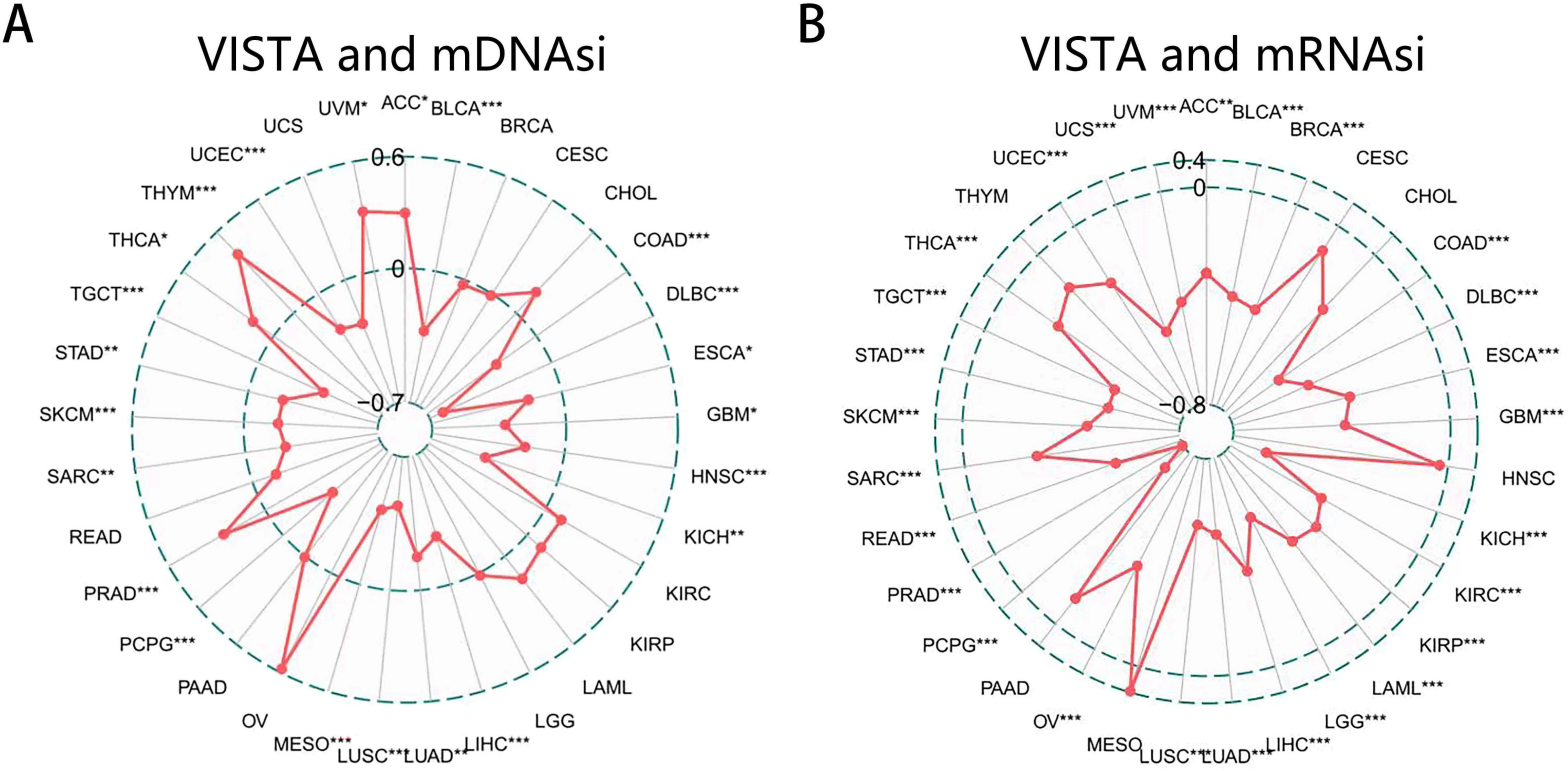
Correlation analysis between VISTA expression and cancer cell stemness. **(A)** The radar chart of the relationship between VISTA expression and mDNAsi (based on epigenetic features). **(B)** The radar chart of the relationship between VISTA expression and mRNAsi (based on gene expression). The points represented the Spearman correlation coefficient. * p < 0.05, ** p < 0.01, *** p < 0.001.

The progression of cancer is accompanied by a gradual loss of differentiation phenotypes as well as the increasingly pronounced stem cell properties which can be used as a predictive biomarker for tumor prognosis (94, 95, 117). One of the major difficulties in cancer treatment lies in its heterogeneity, and even with the successful removal of numerous cancer cells, a small number of cancer stem cells are sufficient to form new tumors. Because the heterogeneity is mostly induced by abnormal cell differentiation and stem cell signaling, understanding the impact of cancer cell stemness will greatly improve the clinical treatment and help to predict the tumor prognosis. The direct assessment of tumor stemness is a little complicated, so we referenced the findings of Malta et al. and applied their scoring method to indirectly assess this feature in each patient (117). In short, two independent stemness indexes, mDNAsi which indicates epigenetic features and mRNAsi which reflected gene expression, were obtained by a multiplatform analysis of the stem cell transcriptome, methylome, and transcription factor binding sites. The closer the mDNAsi or mRNAsi index is to 1, the stronger the degree of cancer cell stemness, which also means that the tumor cells are less differentiated. As we can see, the expression of VISTA was positively correlated with the mDNAsi in ACC (r = 0.248), PRAD (r = 0.216), THCA (r = 0.133), THYM (r = 0.363), and UVM (r = 0.272), while it had negative correlation with the mDNAsi in 17 cancers (**Figure 6A**). We also found that the expression of VISTA was dramatically negatively correlated with mRNAsi in 27 cancers. However, there was no significantly positive association between VISTA and mRNAsi in any type of cancer (**Figure 6B**). We therefore speculated that this result may serve as one of the reasons why VISTA is associated with a good prognosis.

### Correlations between VISTA and DNA/RNA methylation regulatory genes

DNA and RNA methylation are strongly implicated in tumorigenesis and have guiding implications for the development of powerful diagnostic (98, 118). Unlike TMB, stemness, etc., there is no scoring algorithm for methylation and in fact it is difficult to quantify the degree of methylation scored for each patient. However, the genes responsible for methylation in cells are well defined, such as DNA methylation regulatory genes (typically 5mC: DNMT1, DNMT3A, DNMT3B, DNMT3L) (97) or RNA methylation (typically m6A: ALKBH5, FTO, METTL3, METTL14, WTAP) (98), we can roughly estimate the degree of methylation in tumors by assessing the expression of these genes. The relationships between co-inhibitory molecule genes and DNA/RNA methylation regulatory genes were presented in the **Supplementary Tables 7, 8**. As shown in **Supplementary Figure 3**, our results show a correlation between the expression of VISTA and genes associated with DNA/RNA methylation. It may contributed to the hypothesis that it could influence tumor prognosis by altering the methylation levels. Of course, this is a preliminary assumption, by measuring the actual methylation levels in conjunction with tumor expression profiles, further evidence can be provided to support the correlation between VISTA and methylation (119, 120).

### Correlation analysis between VISTA expression and drug sensitivity

**Figure 7:**
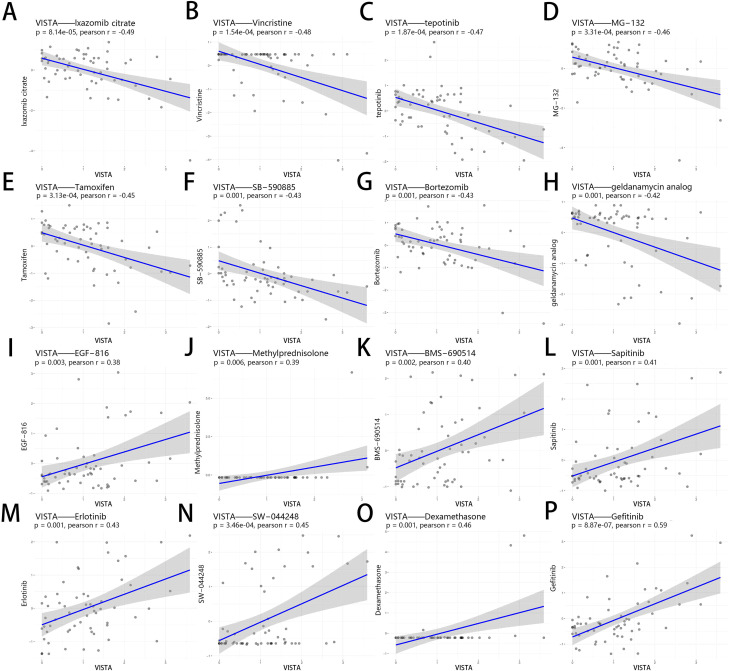
Correlation analysis between VISTA expression and drug sensitivity of anticancer drugs in NCI-60. The drugs shown in the figure were the top 16 drugs with the most significant correlation with VISTA. The 16 drugs, labeled from **(A–P)**, are as follows: Lxazomib citrate, Vincristine, Tepotinib, MG−132, Tamoxifen, SB−590885, Bortezomib, Geldanamycin analog, EGF−816, Methylprednisolone, BMS−690514, Sapitinib, Erlotinib, SW−044248, Dexamethasone, and Gefitinib.

Current strategies targeting co-inhibitory molecules, such as PD1, relies mainly on monoclonal antibodies, with some studies attempting to develop small molecule inhibitors. Since tumor cells can interact with their environment in complicated manners and contribute to an immune suppressive environment that fights against anticancer drugs. ICIs combined with chemotherapy have become one of the research hotpots in the field of tumor immunotherapy to effectively improve response rates of cancer therapies (121). Given that new use of old drugs is a very important development strategy and that the use of drugs in combination with ICIs is currently a crucial research direction, we sought to analyze the relationship between drug sensitivity and co-inhibitory molecules in the NCI60 database, with more than 20,000 drugs (**Supplementary Table 9** and **Supplementary Figures 4A–R**). Here, **Figure 7** illustrated that the expression of VISTA had a negative correlation with the sensitivity to Lxazomib citrate (r = -0.49), Vincristine (r = -0.48), Tepotinib (r = -0.47), MG−132 (r = -0.46), Tamoxifen (r = -0.45), SB−590885 (r = -0.43), Bortezomib (r = -0.43), Geldanamycin analog (r = -0.42). The higher the expression of VISTA, the stronger the drug sensitivity to EGF−816 (r = 0.38), BMS−690514 (r = 0.40), Sapitinib (r = 0.41), Erlotinib (r = 0.43), SW−044248 (r = 0.45), Gefitinib (r = 0.59) (all p <= 0.01). Although Dexamethasone and Methylprednisolone demonstrated a positive correlation with drug sensitivity, these findings were driven by a limited number of data points. Consequently, we have excluded these results from our final analysis to ensure the robustness and reliability of our conclusions. Among the drugs mentioned above, several have already been under clinical trials in combination therapy with ICIs (**Table 1**). Notably, Erlotinib, Dexamethasone and Gefitinib had shown prospective value in combination with PD-1/PD-L1 inhibitors in cancer treatment (122–124). Unfortunately, however, to date, there have been no reports linking VISTA to these drugs. Our analysis suggests that the combination of VISTA and these drugs may also have potential applications.

**Table 1:** A summary of clinical trials of targeted drugs in combination with immune checkpoint inhibitors.

Drugs	ICIs	Targets	Phases	Tumors	Identifier
Erlotinib	IPI or NIV	CTLA4 or PD-1	1	NSCLC	NCT01998126
	NIV	PD-1	1	NSCLC	NCT01454102
	PEMBRO	PD-1	1/2	NSCLC	NCT02039674
Dexamethasone	PEMBRO	PD-1	2	PCM	NCT02880228
	PEMBRO	PD-1	3	MM	NCT02576977
	PEMBRO	PD-1	3	MM	NCT02579863
Gefitinib	DURVA	PD-L1	1	NSCLC	NCT02088112
	PEMBRO	PD-1	1/2	NSCLC	NCT02039674

ICIs, immune checkpoint inhibitors; IPI, Ipilimumab, NIV', Nivolumab; PEMBRO, Pembrolizumab; DURVA, Durvalumab; NSCLC, Non-Small Cell Lung Carcinoma; PCM, Plasma Cell Myeloma; MM, Multiple Myeloma.

### Clinical trials on VISTA

Immunotherapies have enriched the types of available cancer treatments, with the current clinically approved ICIs focusing on PD-1, CTLA4 and PD-L1. Although they offer hope for a cure for many cancer patients, they have many limitations, such as restricted indications for tumor types and only a fraction of patients responding effectively to these agents (61). Novel strategies targeting alternative co-inhibitory molecules including LAG3, TIM3, etc. are therefore proposed (63). Among these, VISTA has been one of the most recently raised inhibitory molecules as a potential target. A growing number of preclinical trials have shown that blocking VISTA enhances the fraction, proliferation, and function of tumor-infiltrating T cells, thus rescuing the TME from the inhibitory state (46). From the results of our analysis and the available clinical studies, it is clear that VISTA has important implications, but there is no comprehensive summary of clinical studies for it. To better understand the potential application of VISTA, we summarized currently undergoing clinical trials in **Table 2**. In a Phase I clinical trial, the safety and efficacy of JNJ-61610588, a fully human IgG1 anti-VISTA mAb, was evaluated in patients with advanced cancer. CA-170, an oral inhibitor targeting both PD-L1/L2 and VISTA, has shown clinical efficacy in phase I and II clinical trials in different advanced solid tumor types (125). Another candidate, CI-8993, is under dose study based on its safety. Since VISTA and PD-1/PD-L1 pathways were proved to have different mechanisms in controlling T cell activation, co-blocking these two signaling pathways shows great prospects in anti-tumor therapy (126), the HMBD-002 (127), a novel anti-VISTA mAb, is being evaluated combined with Pembrolizumab.

**Table 2:** Clinical trials of drugs targeting VISTA in cancer immunotherapy.

Agents	Mechanism of action	Phase	Tumors	Identifier
JNJ-61610588	Anti-VISTA mAb	1	ASTs	NCT02671955
CA-170	Small molecule target VISTA and PD-L1/L2	1	ASTs; Lymphomas	NCT02812875
CI-8993	Anti-VISTA mAb	1	ASTs	NCT04475523
HMBD-002	Anti-VISTA mAb; Or combined with PEMBRO	1	ASTs	NCT05082610

mAb, Monoclonal antibody; ASTs, Advanced solid tumors; PEMBRO, Pembrolizumab.

## Discussion

Co-inhibitory molecules play a vital role in maintaining immune homeostasis by regulating the dynamic of the immune response (128, 129). Unfortunately, however, tumor cells have taken advantage of this mechanism (130) by aberrantly expressing inhibitory ligands to evade immune surveillance. As a result, ICIs treatment, which restores the activity of immune cells, represents one appealing therapeutic strategy that has provided long-term remission for cancer patients. However, only a limited number of patients respond to ICIs therapy, such as CTLA4 and PD-1 inhibitors. A substantial proportion of patients do not respond to ICIs, with the response rate to anti-PD-1 therapy being approximately 25% even in melanoma, which demonstrates the highest response rates among cancers (131). Even if patients initially respond to ICIs, they may develop resistance over time, and the specific mechanisms underlying this resistance remain poorly understood (132). At the same time, immune-related adverse events (irAEs) associated with ICIs therapy appear to be inevitable. Inflammatory reactions affecting the skin, gastrointestinal tract, liver, endocrine organs, and lungs are the most common, and they may even involve multiple organs (133). This has prompted in-depth research into alternative co-inhibitory molecules and combination therapies, with the aim of expanding the patient population and effectiveness of immunotherapy, promoting personalized treatment, and minimizing severe irAEs.

In the present study, we conducted a comprehensive pan-cancer analysis to evaluate the significance of the co-inhibitory molecules across various types of cancers. In the differential expression analysis, we found that the expression of these molecules varied from each other. It showed that there were definite trends of these molecules, especially CTLA4, LAG3, PD-1, TIGIT, TIM3, CD80, CD155, VEGFA, towards high expression in tumor cells, whereas VISTA tended to have lower expression levels compared with corresponding normal cells intriguingly. Abundant evidence supports the findings from our analysis. For example, Liu et al. performed a study to analyze the clinical implications of PD-1 and CTLA4 expression and their results for differential expression are consistent with ours (134). Similarly, by flow cytometry, TIGIT protein has been detected to be highly expressed in human renal cell carcinoma, lung, breast, and ESCA (17, 63, 135, 136). Besides, according to Anderson et al., TIM3 is usually highly expressed in cancer compared with normal tissues and enhances suppression of protective immunity (25). In our research, the expression level of FGL1 appeared to be extremely low, except in CHOL, LIHC and corresponding normal tissues, which might be due to the fact that FGL1 is secreted out at these locations (28). NRP1 and VEGFA had high expression levels in KIRC, a result that could also be confirmed by Morin et al. (137) and Wang et al. (138), respectively. Our observations of VISTA are similar not only to those of Huang et al. (139) but also to previous reports showing that VISTA is less expressed in BRCA, COAD and STAD (140, 141), while more expressed in KIRC when compared to normal tissues (142). The differential expression of co-inhibitory molecules across different tumors helps us better understand patient resistance. In fact, this suggests that early screening for co-inhibitory molecule targets in patients is crucial.

Therefore, to benefit a larger number of patients, combination therapies should be considered. In studying the correlation between co-inhibitory molecules, we noted a number of combinations with high correlation scores, such as TIGIT and PD-1 (r = 0.81), LAG3 and PD-1 (r = 0.77). Literature research reveals that many of these combinations are being or warrant further study, such as clinical studies that are attempting to use co-blockade to suppress tumors. For instance, CD8^+^ T cells expressing TIGIT usually co-express PD-1, and their simultaneous blockade *in vitro* increases cytokine production (18) and leads to significant reversal of tumor growth (17). Current research suggests that CD226 is a key locus for the combined blockade effects of TIGIT and PD-1 therapies. Both molecules can mediate tumor immune evasion by inhibiting the activation function of CD226 (143). However, the specific mechanisms are different: TIGIT primarily exerts its effects by competitively binding to the CD226 ligand, CD155, whereas PD-1 exerts its effects by recruiting SHP2 to dephosphorylate CD226 (144). In addition, it has also been reported that LAG3 and PD-1 are widely co-expressed on infiltrating CD4^+^ and CD8^+^ T cells in transplantable tumors, with a potential therapeutic advantage using co-blockade (106, 145). Notably, we found a remarkably positive correlation between PD-1 and LAG3 in SKCM and UVM (ranked first and second, respectively). PD-1 and LAG-3 are considered to cooperate on CD8+ T cells, promoting T cell exhaustion and limiting anti-tumor immune responses by regulating the Thymocyte selection-associated HMG box protein (TOX) gene. Their deletion significantly enhances the anti-tumor activity of CD8+ T cells (146). The FDA has approved Opdualag, containing Relatlimab (anti-LAG3) and Nivolumab (anti-PD-1), for the treatment of patients with unresectable or metastatic melanoma, based on the prolongation of progression-free survival (109). On the other hand, the combinations of low correlation are of equal interest, as it may imply the non-redundancy between their functions. Our results showed a weak correlation between VISTA and PD-1/PD-L1, and they are proven to play distinct roles in controlling T cell activity (126). More importantly for this non-redundant role, synergistic effects of combined blockade have been demonstrated (147), i.e., patients who do not respond to PD-1/PD-L1 inhibitors might benefit from VISTA blockade (46). Therefore, applying intermolecular correlation analysis to guide the development of novel combination therapeutic strategies can be a reasonable assumption.

We also explored the association between co-inhibitory molecules and tumor prognosis as well as immune cell infiltration. The infiltrating immune cells calculated by different methods are not identical, which might be due to the different algorithms and the availability of samples. CD8^+^ T cells are generally considered to have a major contribution to antitumor immunity (148), as they are the key immune cells that kill cancer cells presenting MHC class I molecules (114). Activated CD8^+^ T cells express IRs extensively on their surface, thus inhibiting the activation of otherCD8^+^ T cells or causing their exhaustion. In recent years, in addition to CD8^+^ T cells, the importance of other immune cells in antitumor immunity has been gradually emphasized (149–151). Although it is generally believed that co-inhibitory molecules are involved in T cell failure and negatively modulate the immune response, we found that their roles as biomarkers in prognosis were not all negative, but highly variable. This may suggest that they have different roles in different type of tumor. When combining survival and immune cell infiltration analysis, it is noteworthy that VISTA was positively associated with prognosis as well as immune cell infiltration in most cancers, this finding is further supported by existing studies (152, 153). This result not only indicates that immune cell infiltration in TME plays a critical role in the way that VISTA affects survival, but also suggests that VISTA may serve as a positive prognostic biomarker in specific cancer types and potentially function in a manner analogous to co-stimulatory molecules.

As we know, VISTA has been identified as an inhibitory receptor (46) and is associated with poor prognosis in patients with cancers such as PAAD (154), colorectal cancer (155), oral squamous cell carcinoma (156), et al. Meanwhile, other studies have found that VISTA can bind to LRIG1 on CD8+ T cells to exert inhibitory effects (55). However, there is also compelling evidence that VISTA is positively correlated with prognosis and functions as a co-stimulatory molecule (157–160). For instance, high expression of VISTA is strongly related to good prognosis in ESCA (157), HCC (158), NSCLC (159), and high-grade serous ovarian cancer (160). Furthermore, VISTA is significantly correlated with the infiltration of CD8^+^ T cells, indicating that VISTA may influence potential pathways in TME that recruit T cell, which in turn attacks tumor cells (158). Previous studies have also observed that the overexpression of VISTA in human monocytes or macrophages induces the secretion of various cytokines, while also acting as a ligand to stimulate T cell responses (161). Moreover, we observed a negative correlation between VISTA and cancer cell stemness, which is generally recognized as an indicator of poor survival. These findings may partly responsible for the positive relationship of VISTA with OS, DSS, PFI in cancers (110). Considering all these findings, the function of VISTA in tumors is more complex than initially expected, and at the very least, it should not be regarded as uniform, as our results shows.

Although we speculate that the VISTA plays a different role in TME and may act as a positive prognostic biomarker in some cancers, we cannot easily determine whether VISTA has the potential to be a co-stimulatory receptor due to the limited studies. Anyway, according to previous studies, it is undeniable that VISTA can be an effective or at least a potential target for cancer immunotherapy, such as melanoma (Isabelle et al. achieved optimal efficacy with a combination therapy using an anti-VISTA mAb and a peptide-based cancer vaccine) (46), PAAD (154, 162, 163), Glioblastoma (49), fibrosarcoma (45), squamous carcinoma (164), etc. (139). Moreover, dual blockade of VISTA with other co-inhibitory molecules has also yielded remarkable outcomes (160, 164–166). Combined blockade of VISTA with PD-1 has been reported to reduce tumor size, improve survival (126, 167), and significantly enhance the recruitment of CD8^+^ T cells (164). Meanwhile, dual blockade of VISTA and CTLA4 can markedly inhibit tumor progression and Treg cells recruiting (164). More importantly, our findings regarding the correlation of VISTA with TMB, MSI, cancer cell stemness, as well as DNA/RNA methylation regulatory genes may accelerate the application of VISTA as a target in individualized therapy. Zaravinos and colleagues have revealed that MSI-H colorectal cancers expressing high VISTA levels responded more intensively to anti-VISTA immunotherapy compared to tumors with stable microsatellites (168). Besides, since an increase in cancer cell stemness would decrease the effectiveness of anti-PD-L1 mAb treatment for certain cancers (GBM, LUSC, HNSC, and BLCA) (117), it’s reasonable for us to speculate on the significance of the dual assessment of cancer cell stemness and VISTA expression based on the homology of VISTA and PD-L1 (41, 42), In addition, the expression of DNA/RNA Methylation genes is another means by which tumor cells evade immune surveillance. An effective strategy to address this issue may be the combination of anti-VISTA mAb and methylase inhibitors (169). As mentioned above, VISTA is an immunotherapeutic target that holds promise not only in monotherapy but also in combination therapy strategies. However, it is important to recognize that combination therapies targeting immune checkpoints often lead to more severe irAEs, a phenomenon that has been widely observed in patient cohorts receiving anti-CTLA-4 and anti-PD-1/PD-L1 combination therapy. Some irAEs can even be fatal, particularly those related to the heart (170, 171). Therefore, it is crucial to conduct more cautious studies on co-inhibitory molecules, delve deeper into their signaling pathways, understand their synergistic mechanisms, and investigate the potential causes of irAEs development.

RNA as an Intermediary of Gene Expression, the approach of analyzing total RNA data from pan-cancer samples provides a means to characterize the TME, particularly in the context of the expanding volume of transcriptomic data available today (172). Current research has shown that RNA can serve as a biomarker for the preliminary diagnosis of patients. Total mRNA from tumors can characterize heterogeneity both between different tumors and within the same tumor type, and it can also serve as a method for predicting clinical outcomes in cancer patients (172–176), or be used to investigate the mechanisms of inhibitory molecules (177). With the further exploration of RNA transcriptional information through techniques such as ribosome profiling (Ribo-Seq), it is anticipated that these advancements will provide deeper insights for the characterization and treatment of tumors (178). However, we must acknowledge that there are certain limitations in our study that require further resolution. Firstly, as previously mentioned, the extensive heterogeneity across different cancers, and even within the same cancer type, both can lead to varying gene expression levels (179). Although pan-cancer analysis provides a broad perspective on the expression patterns of co-inhibitory molecules, features such as TMB, MSI, and co-regulatory molecules specific to certain cancers may be overlooked (180). Validation in patient cohorts specific to certain tumor types is helpful for the translation of research findings, as observed in our results, where the expression levels of VISTA varied across different tumors. We also observed that mRNA expression data will be affected by the composition of different cell types, particularly the infiltration of immune cells, this influence may confound our interpretation of the results. The integration of single-cell RNA sequencing helps address these challenges, as its high-resolution capabilities allow for the precise identification of cellular composition, subtypes, cellular heterogeneity, and molecular expression patterns within specific tumors (181). Furthermore, single-cell data can reveal the co-expression of inhibitory receptors and their synergistic interactions with other proteins, providing valuable insights into the underlying molecular mechanisms (182). Another issue is that our study relies on transcriptomic expression data from tumor tissues. Although mRNA data provide valuable insights into gene expression, they have inherent limitations in reflecting the true protein expression levels, cellular localization, and functionality. Generally, although there is often a clear positive correlation between the mRNA and protein abundance of different genes, this correlation can be weakened due to differential translation, protein degradation, and buffering mechanisms (183). Constructing a map of direct protein-protein interactions can provide more robust evidence, particularly when investigating the role of inhibitory receptors in the tumor microenvironment. As demonstrated in the study by Shilts et al., high-throughput screening of surface proteins, combined with single-cell profiling, was used to construct a surface protein interaction network of the human immune system (184). We also recognize that using multidimensional data to characterize samples may provide more valuable insights for the research. Exploring circulating DNA/RNA in patients may be a promising direction for further research (185). Unlike tumor tissue biopsies, circulating nucleic acids can serve as biomarkers for real-time assessment of a patient’s survival status through repeated sampling and continuous monitoring (186, 187). This feature not only enhances the sensitivity of the data but also offers new perspectives, such as exploring the relationship between the expression of co-inhibitory molecules and tumor metastasis (188). In conclusion, it is hoped that these limitations can be addressed in future studies.

## Conclusion

In summary, the results of differential expression analysis, correlation analysis, and drug sensitivity analysis suggest that CTLA4, PD-1, TIGIT, LAG3, TIM3, NRP1, VISTA, CD80, CD86, PD-L1, PD-L2, PVR, PVRL2, FGL1, LGALS9, HMGB1, SEMA4A, and VEGFA are associated with tumor prognosis and immune cell infiltration. Therefore, we believe that they are hopefully to serve as prognostic biomarkers for certain cancers. In addition, our analysis indicates that VISTA plays a complex role and its expression is related to TMB, MSI, cancer cell stemness, DNA/RNA methylation, and drug sensitivity. These findings may provide the basis for VISTA to become a promising target, thus driving the development of novel strategies for tumor immunotherapy, especially individualized and combination therapy.

## Data Availability

The original contributions presented in the study are included in the article/**Supplementary Material**. Further inquiries can be directed to the corresponding author.

## Glossary

CTLA4: Cytotoxic T lymphocyte-associated antigen-4
PD-1: Programmed death receptor-1
DCs: dendritic cells
PD-L1: Programmed cell death ligand 1
PD-L2: Programmed cell death ligand 2
TIGIT: T cell Ig and ITIM domain
NK: Natural Killer
Treg cell: regulatory T cell
LAG3: Lymphocyte-activation gene 3
MHC: Major Histocompatibility Complex
FGL1: fibrinogen-like protein 1
TNF-α: tumor necrosis factor alpha
IFN-β: interferon-β
TIM3: T cell immunoglobulin and mucin-domain containing-3
Tc cell: T cytotoxic cell
HMGB1: high mobility group box 1
NRP1: Neuropilin-1
VEGF: vascular endothelial growth factor
VISTA: V-domain Ig-containing suppressor of T cell activation
VSIG-3: V-set and immunoglobulin domain containing 3
PSGL-1: P-selectin glycoprotein ligand-1
ICIs: immune checkpoint inhibitors
mAb: monoclonal antibody
FDA: US Food and Drug Administration
SCLC: Small-Cell Lung Cancer
NSCLC: Non-Small Cell Lung Carcinoma
MPM: malignant pleural mesothelioma
HCC: Hepatocellular Carcinoma
ACC: Adrenocortical carcinoma
BLCA: Bladder urothelial carcinoma
BRCA: Breast invasive carcinoma
CESC: Cervical squamous cell carcinoma and endocervical adenocarcinoma
CHOL: Cholangiocarcinoma
COAD: Colon adenocarcinoma
DLBC: Lymphoid Neoplasm Diffuse Large B-cell Lymphoma
ECSA: Esophageal carcinoma
GBM: Glioblastoma multiforme
HNSC: Head and Neck squamous cell carcinoma
KICH: Kidney Chromophobe
KIRC: Kidney renal clear cell carcinoma
KIRP: Kidney renal papillary cell carcinoma
LAML: Acute Myeloid Leukemia
LGG: Brain Lower Grade Glioma
LIHC: Liver hepatocellular carcinoma
LUAD: Lung adenocarcinoma
LUSC: Lung squamous cell carcinoma
MESO: Mesothelioma
OV: Ovarian serous cystadenocarcinoma
PAAD: Prostate adenocarcinoma
PCPG: Pheochromocytoma and Paraganglioma
PRAD: Prostate adenocarcinoma
READ: Rectum adenocarcinoma
SARC: Sarcoma
SKCM: Skin Cutaneous Melanoma
STAD: Stomach adenocarcinoma
TCTG: Testicular Germ Cell Tumors
THCA: Thyroid carcinoma
THYM: Thymoma
UCEC: Uterine Corpus Endometrial Carcinoma
UCS: Uterine Carcinosarcoma
UVM: Uveal Melanoma
ASTs: Advanced solid tumors
TCGA: The Cancer Genome Atlas
TIMER: Tumor Immune Estimation Resource
FDR: False Discovery Rate
TPM: Transcripts Per Million
OS: Overall Survival
PFI: Progression-Free Interval
DSS: Disease-Specific Survival
HR: hazard ratio
TMB: Tumor Mutation Burden
MSI: Microsatellite Instability
SSR: simple sequence repeat
5mC: 5-methylcytosine
m6A: N6-methyladenosine
ALKBH5: Human AlkB homolog H5
FTO: Fat Mass and Obesity-associated protein
METTL3: Methyltransferase like 3
METTL14: Methyltransferase like 14
WTAP: Wilms tumor 1-associated protein
TME: tumor microenvironment
FC: fold change
IPI: Ipilimumab
NIV: Nivolumab
PEMBRO: Pembrolizumab
DURVA: Durvalumab
PCM: Plasma Cell Myeloma
MM: Multiple Myeloma
