# Supplementary material for: Pan-cancer analysis of co-inhibitory molecules revealing their potential prognostic and clinical values in immunotherapy
Source: Front Immunol. 2025 Mar 24;16:1544104. doi: 10.3389/fimmu.2025.1544104 (PMC11973099; doi:10.3389/fimmu.2025.1544104)

# Supplementary Fig. 1

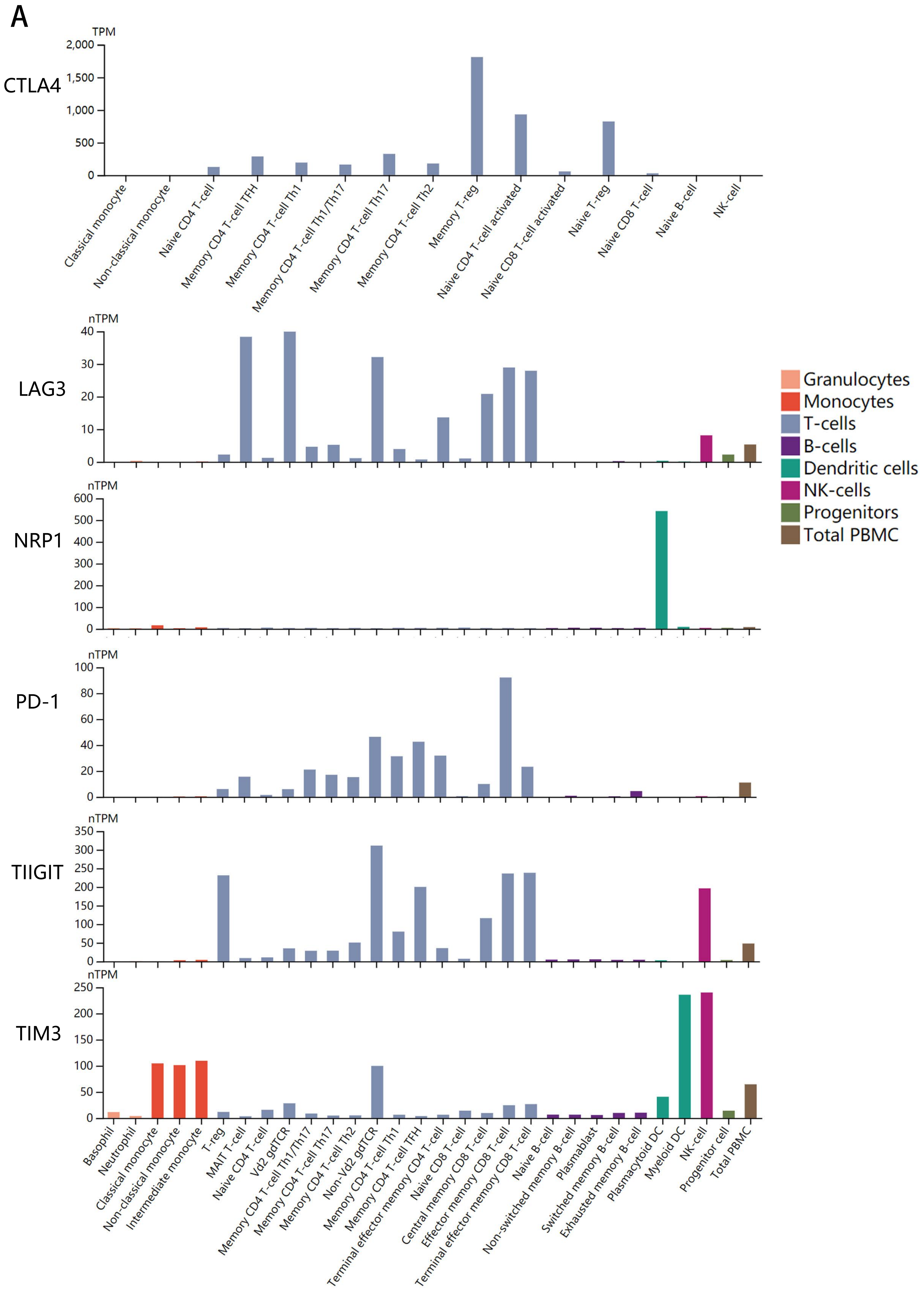

# Supplementary Fig. 1

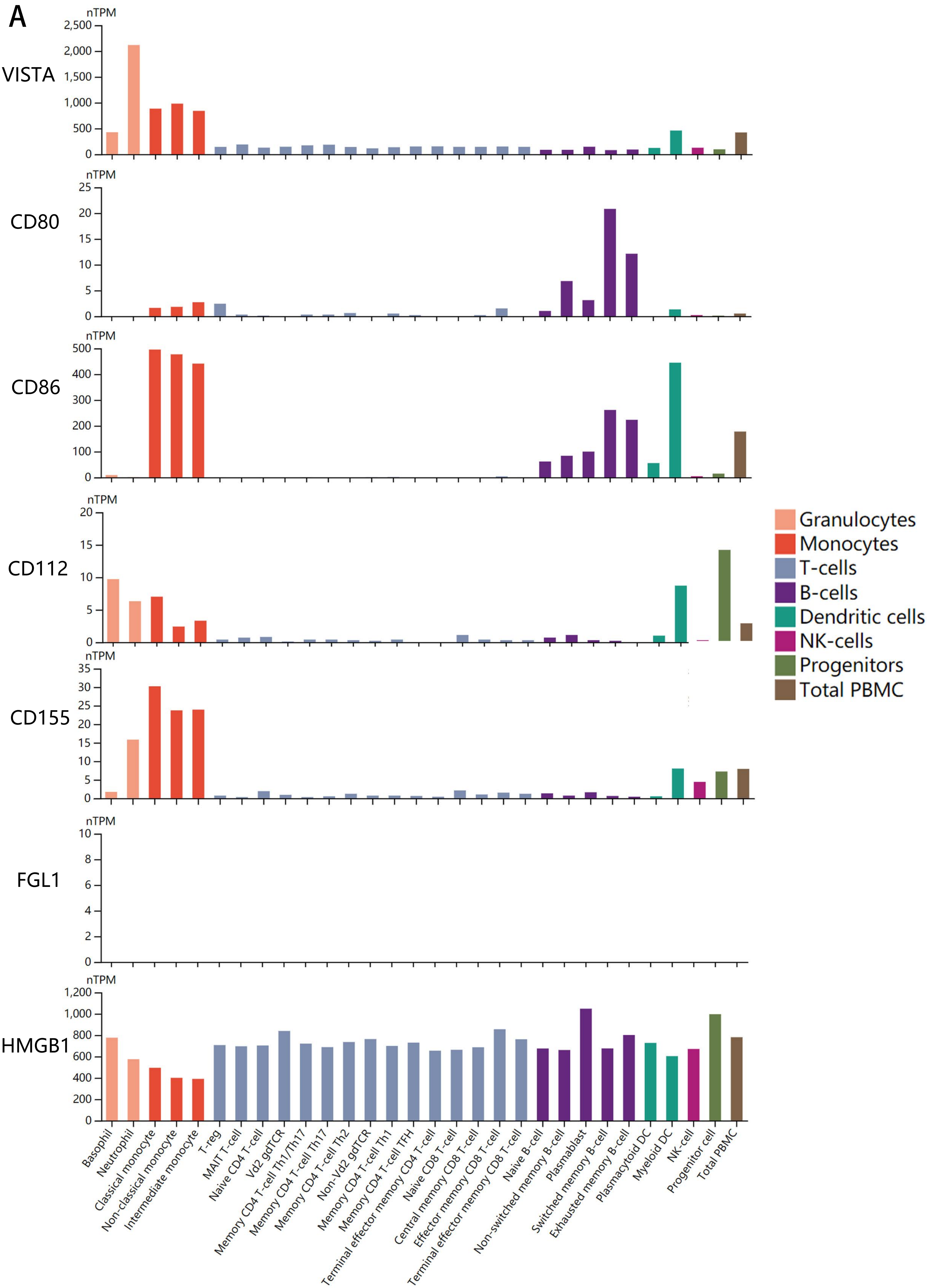

## Supplementary Fig. 1

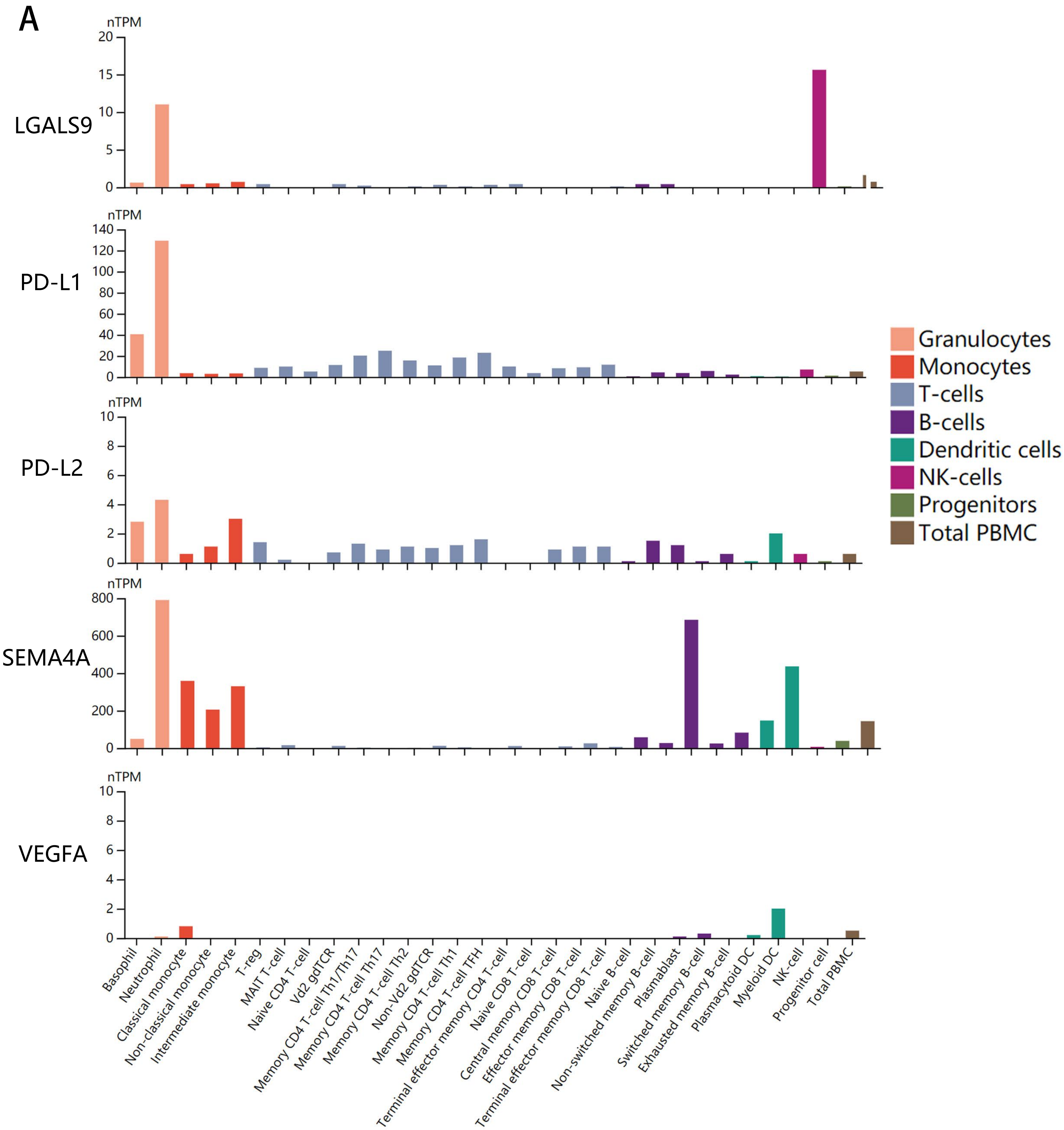

Supplementary Fig. 1

B

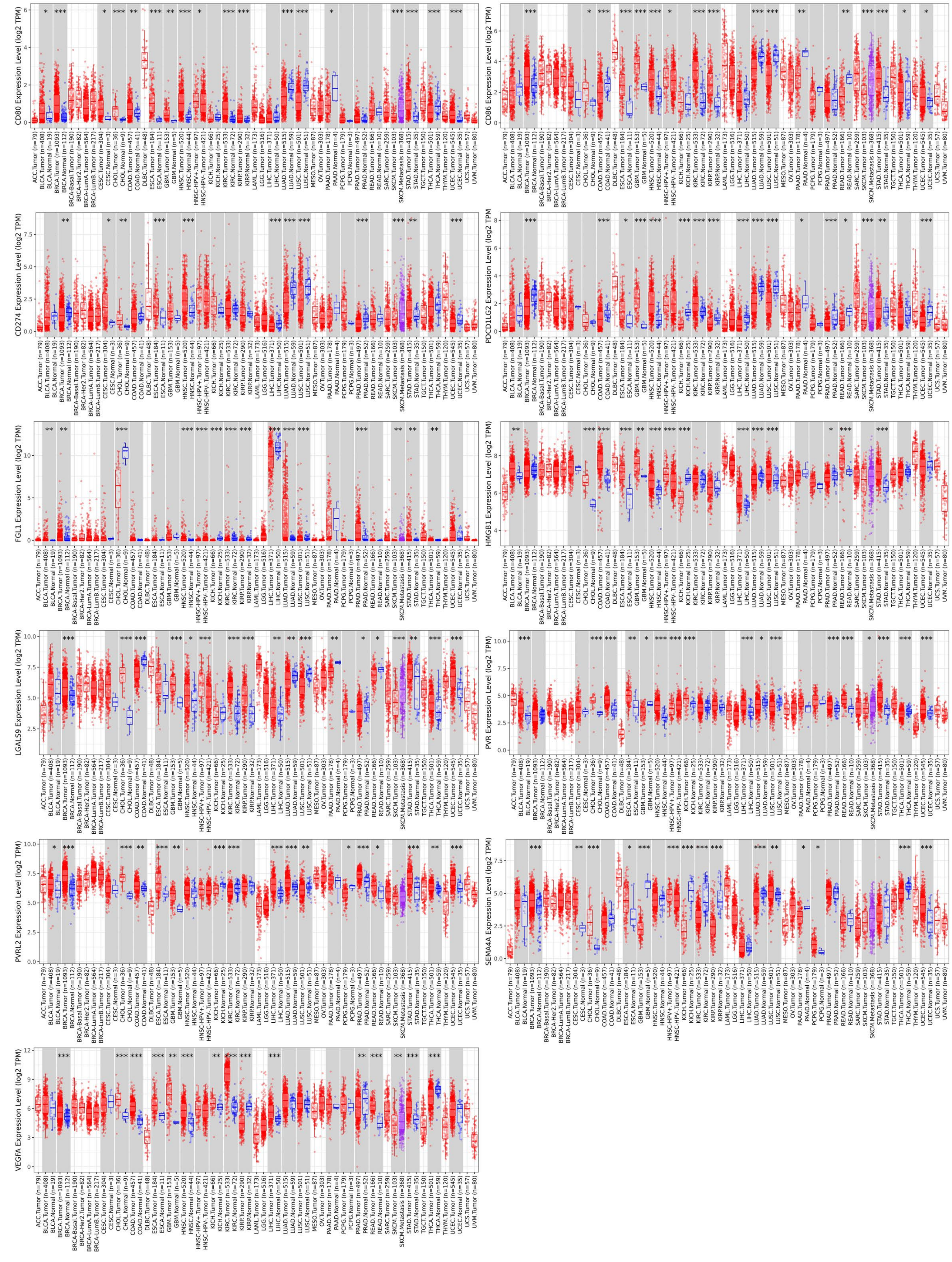

# Supplementary Fig. 2

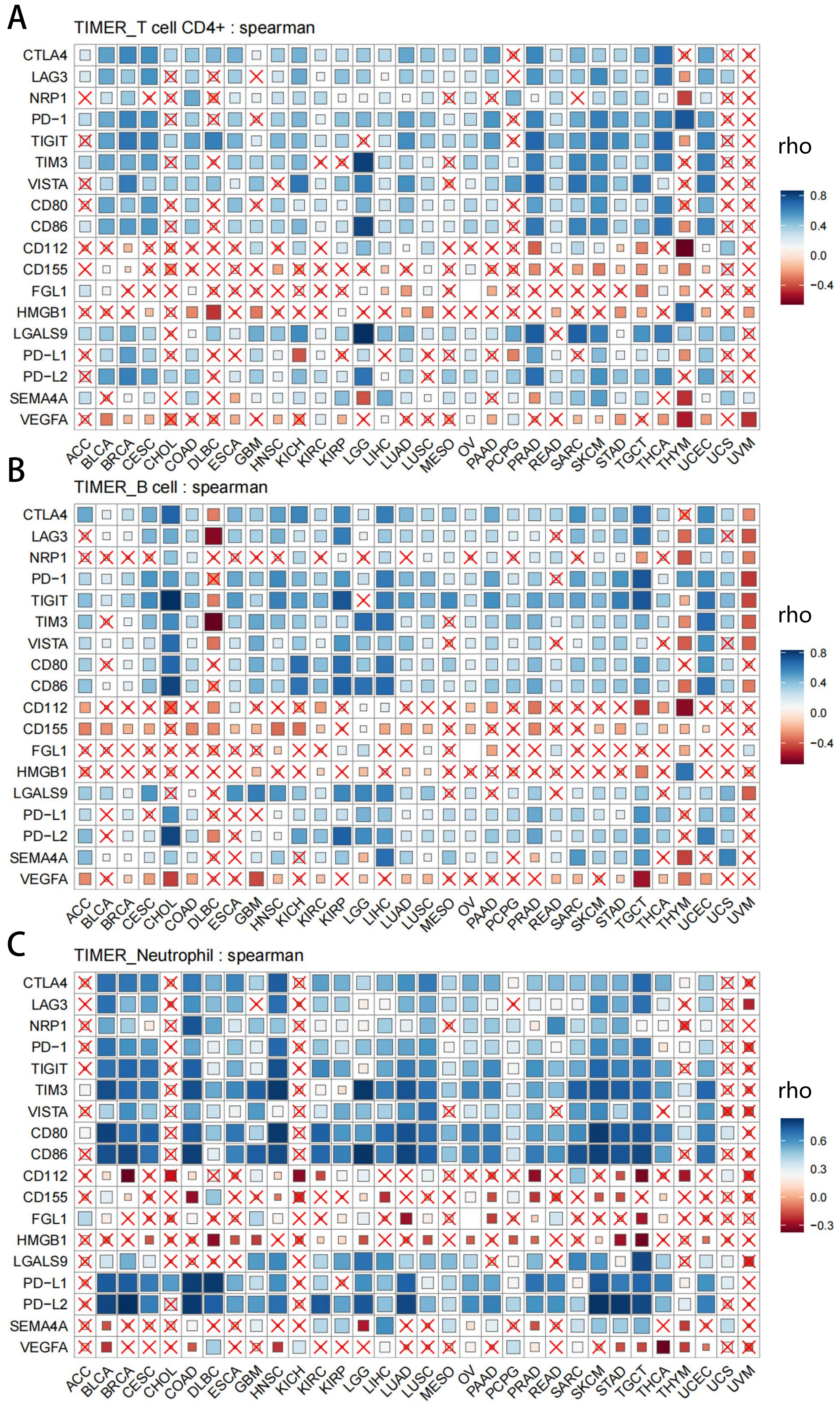

Supplementary Fig. 2

D

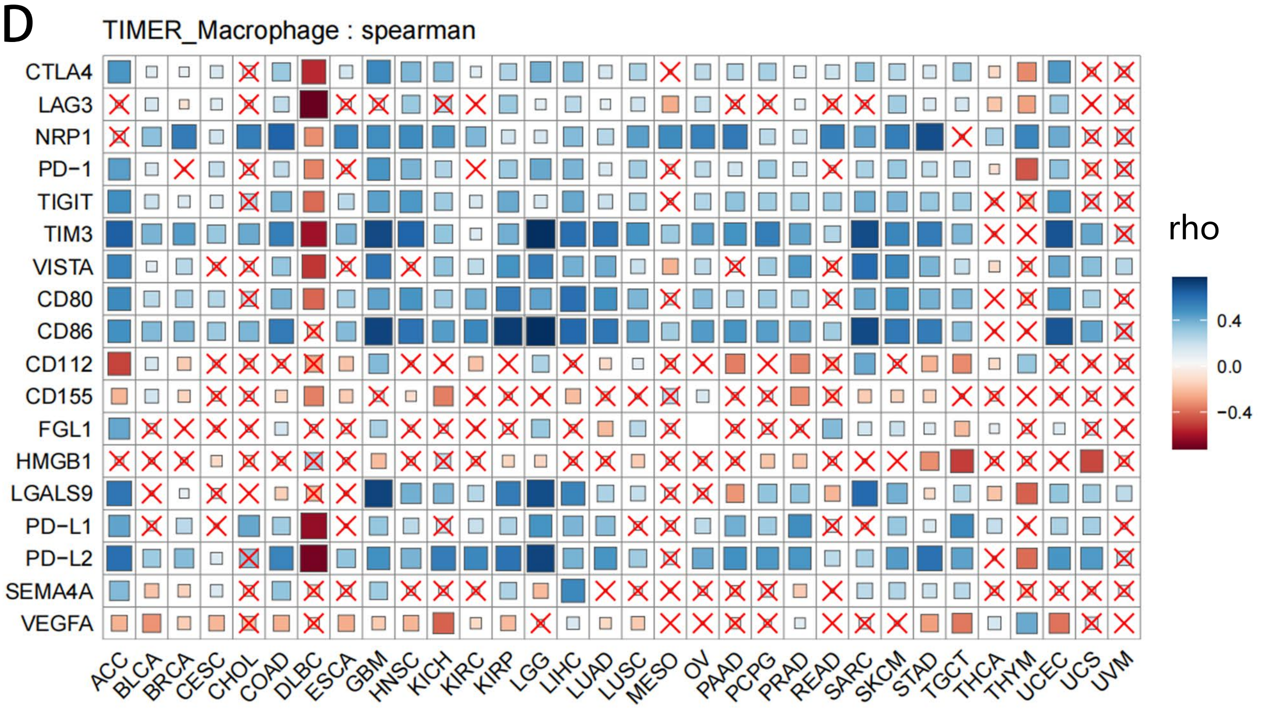


E

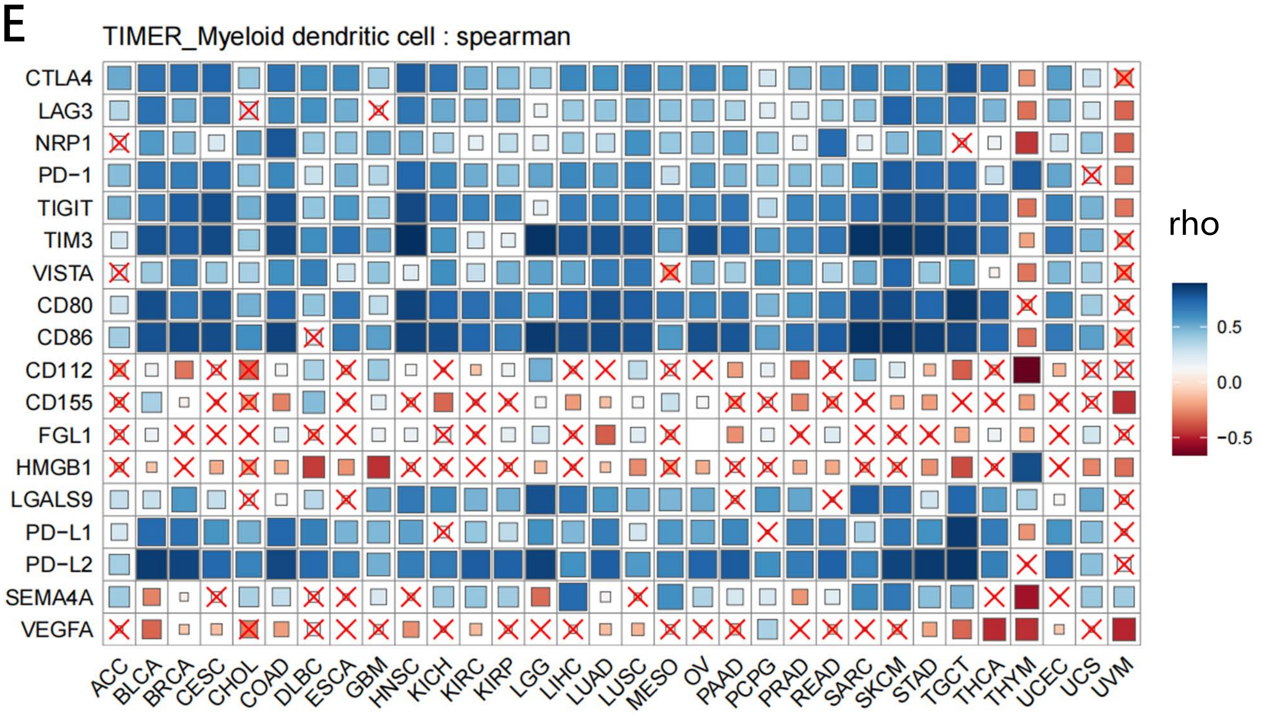

# Supplementary Fig. 3

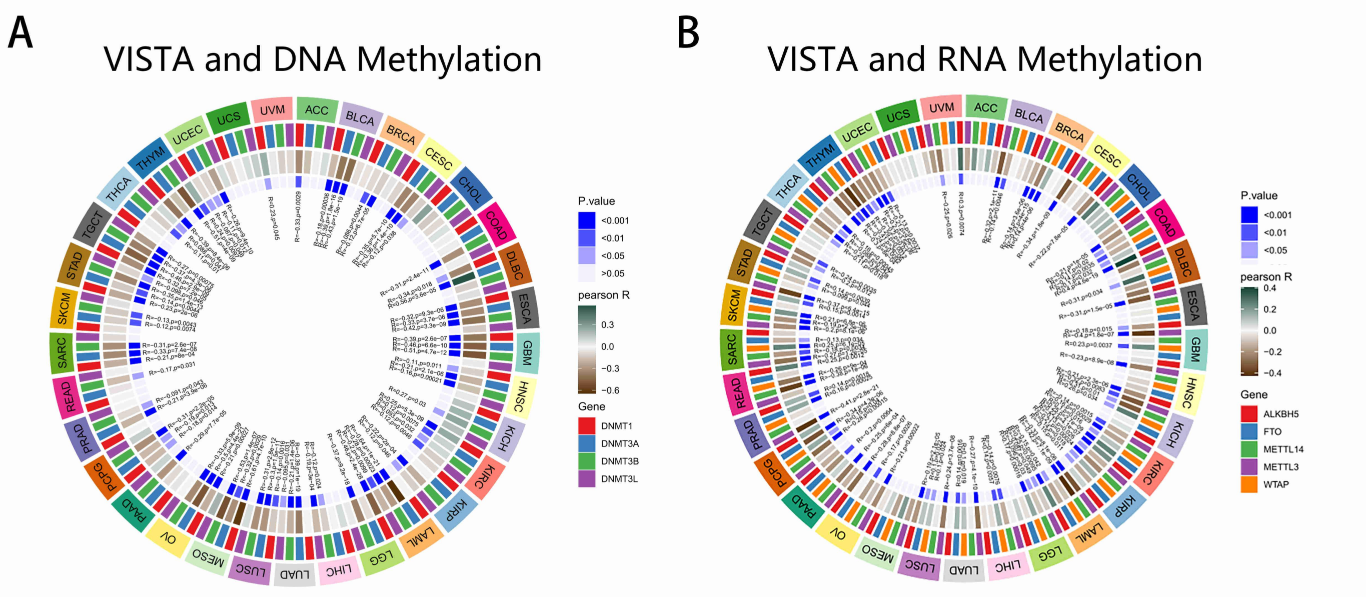

# Supplementary Fig. 4

## A CTLA4

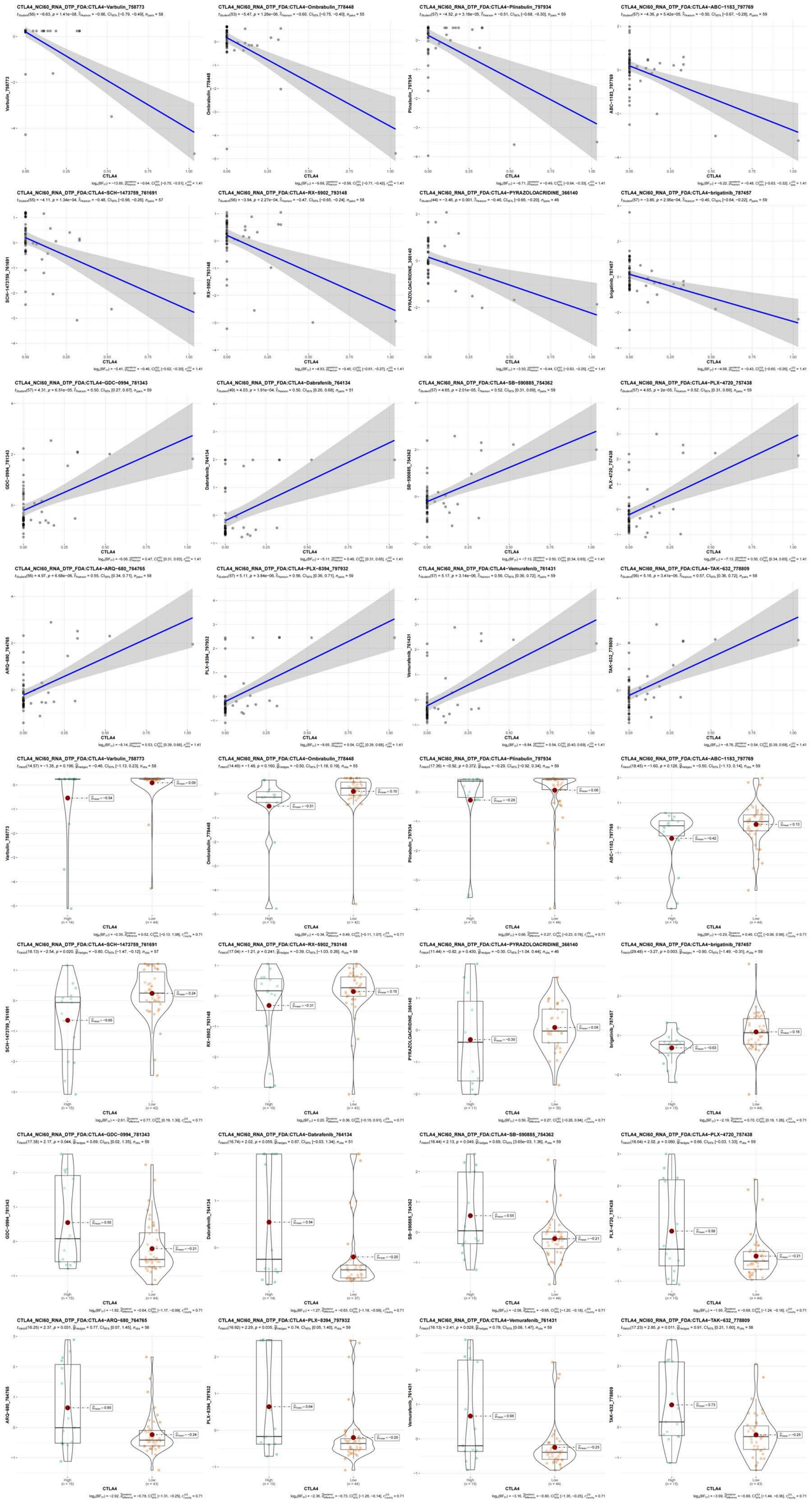

# Supplementary Fig. 4

## B LAG3

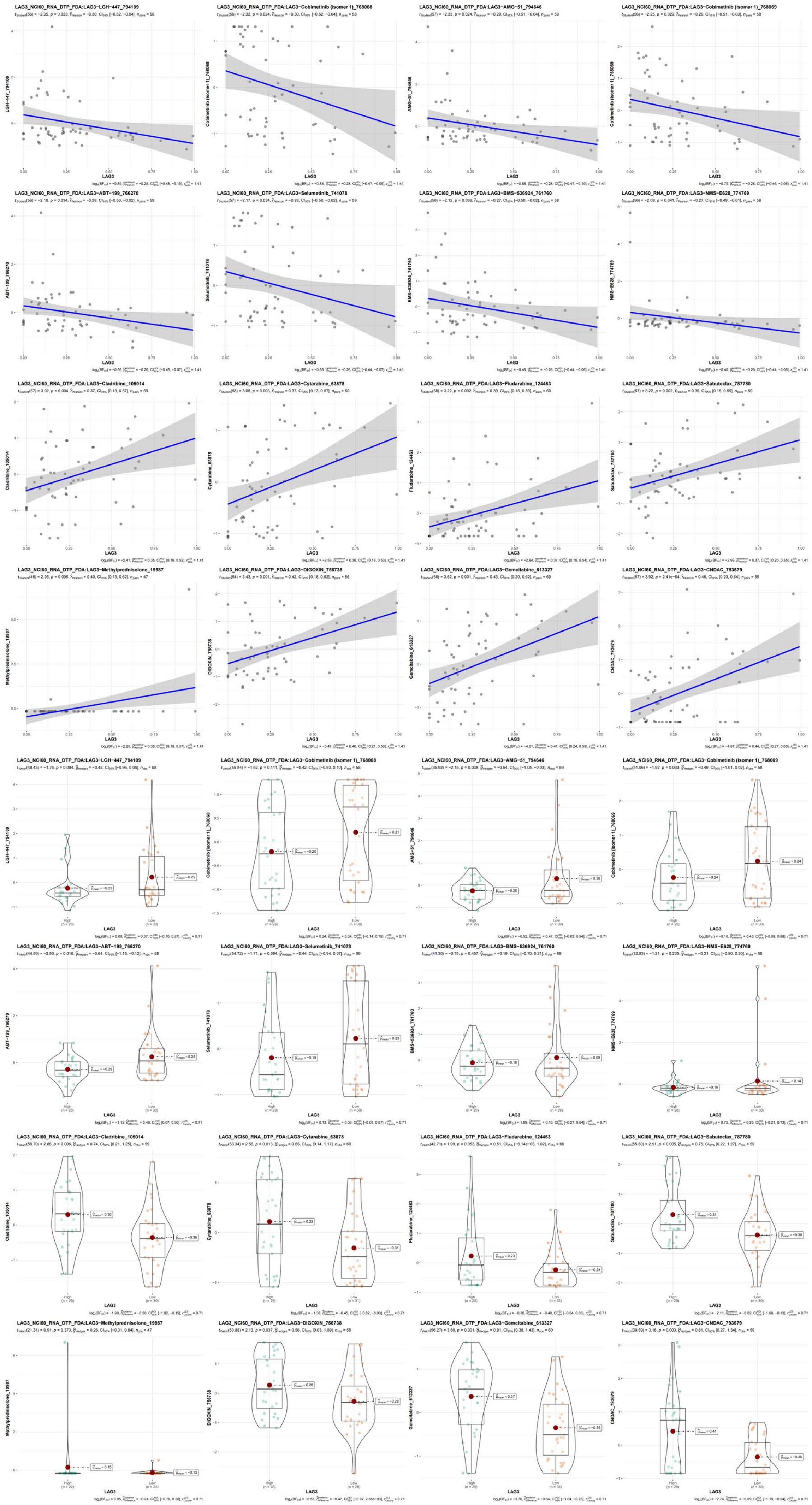

# Supplementary Fig. 4

## C NRP1

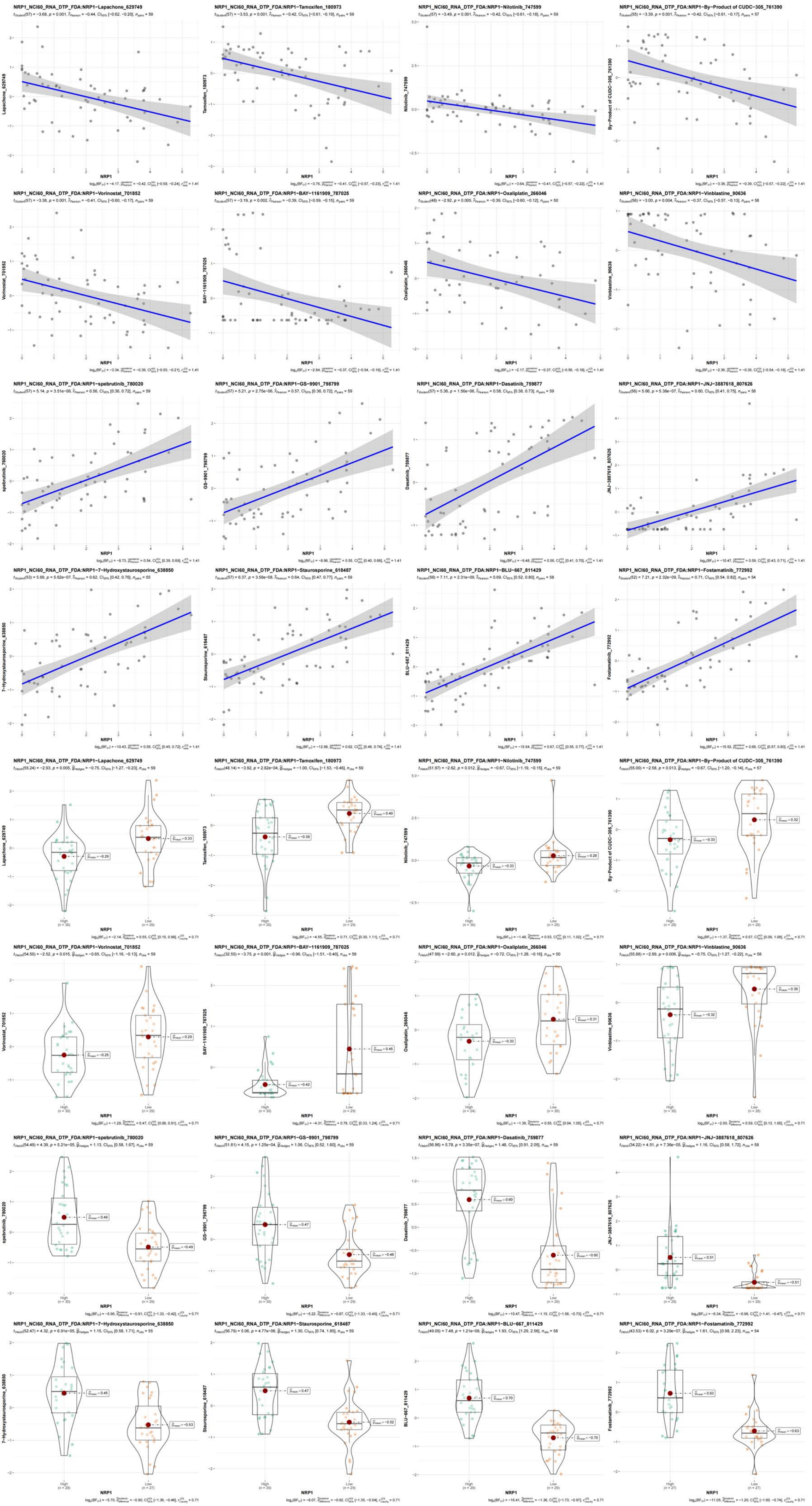

# Supplementary Fig. 4

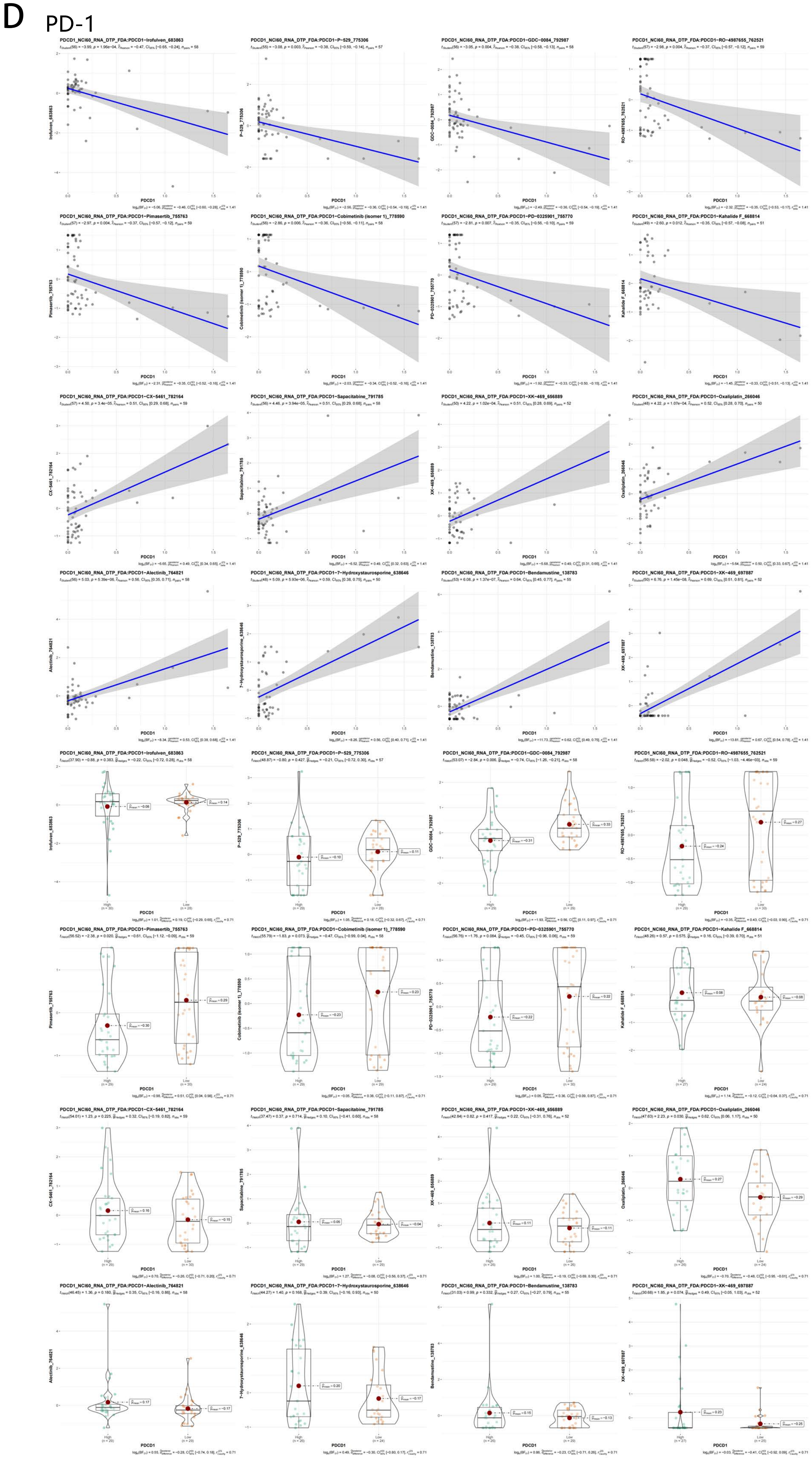

# Supplementary Fig. 4

E TIGIT

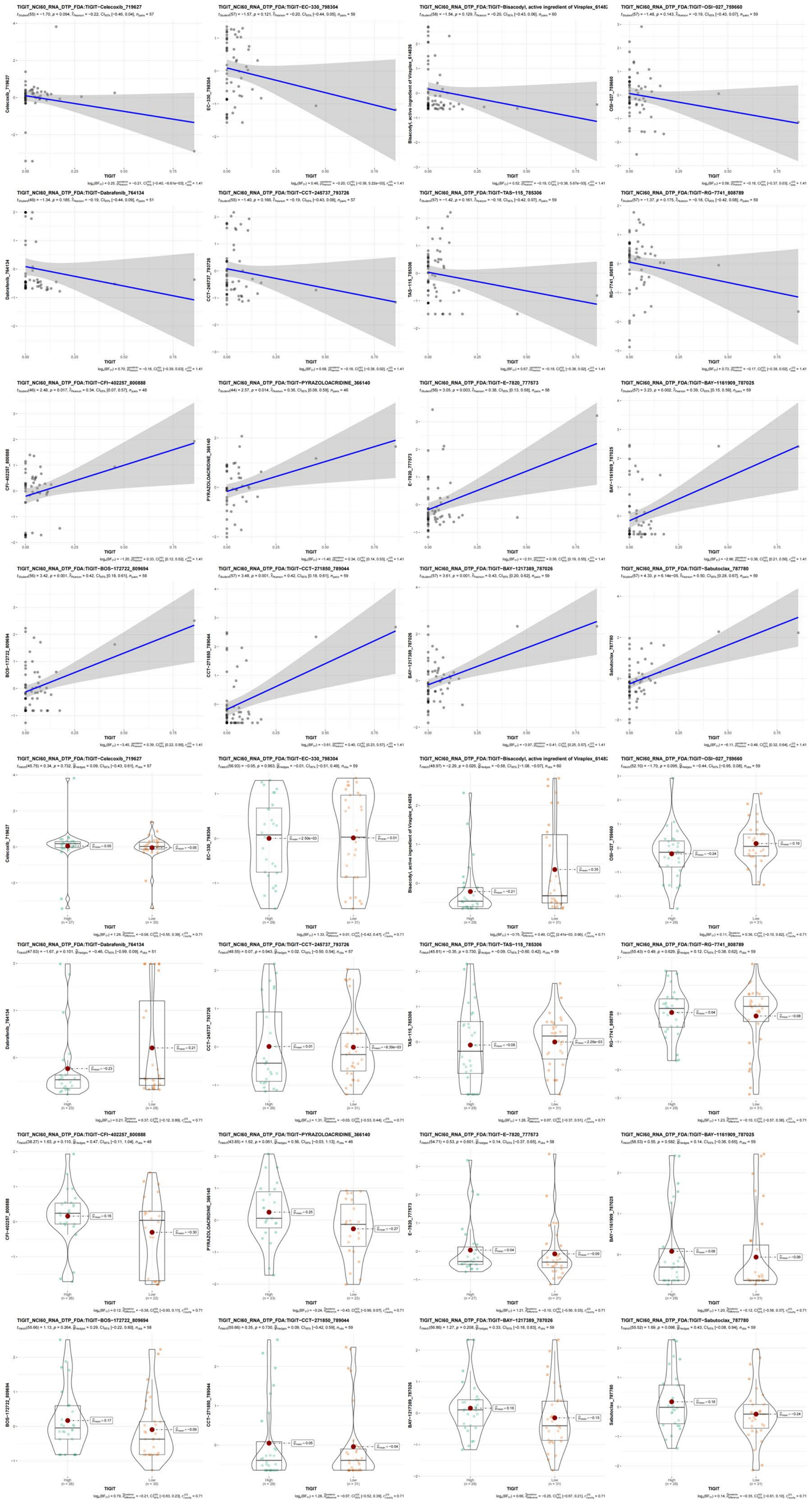

# Supplementary Fig. 4

F TIM3

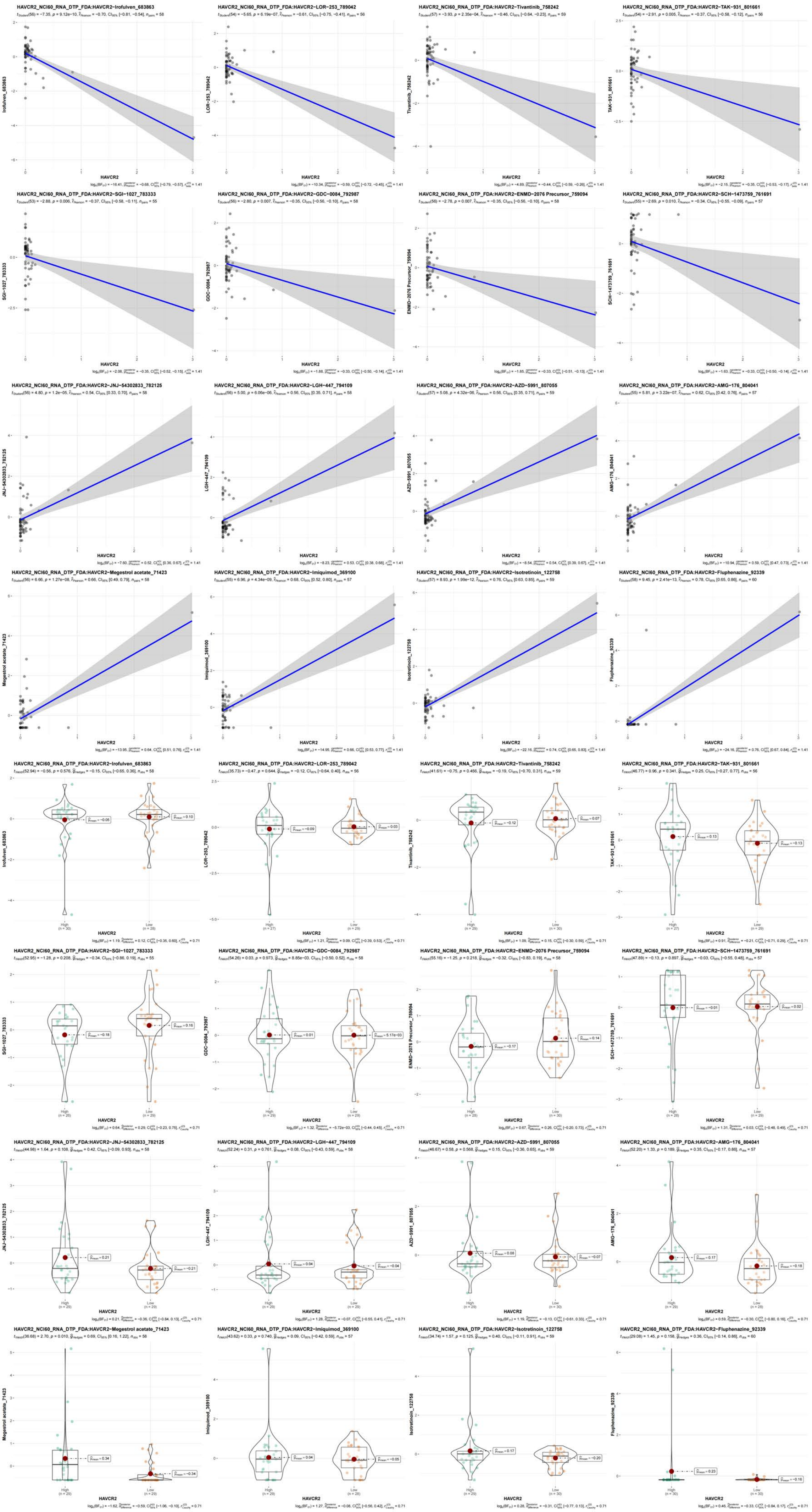

# Supplementary Fig. 4

## G VISTA

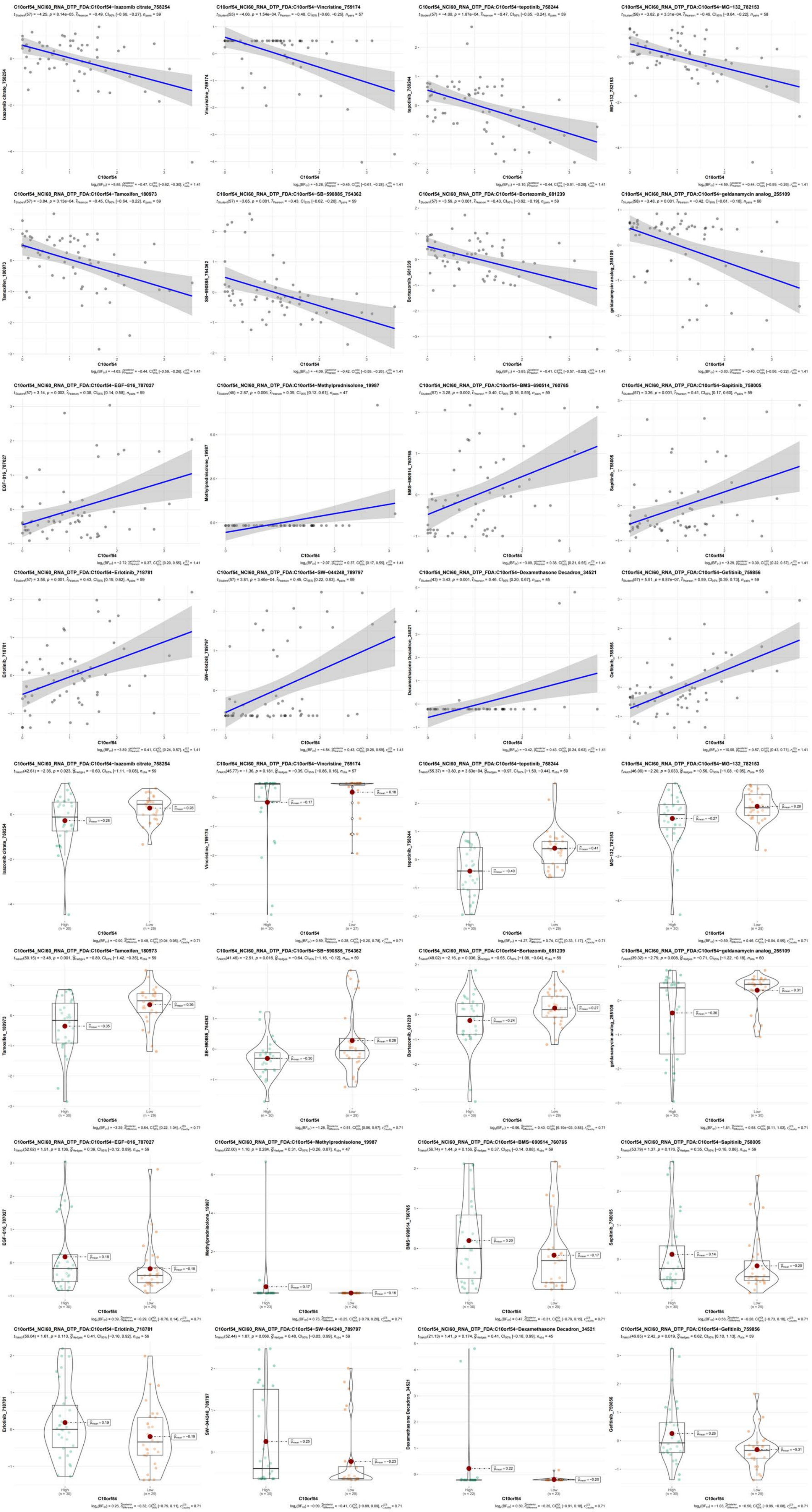

# Supplementary Fig. 4

H CD80

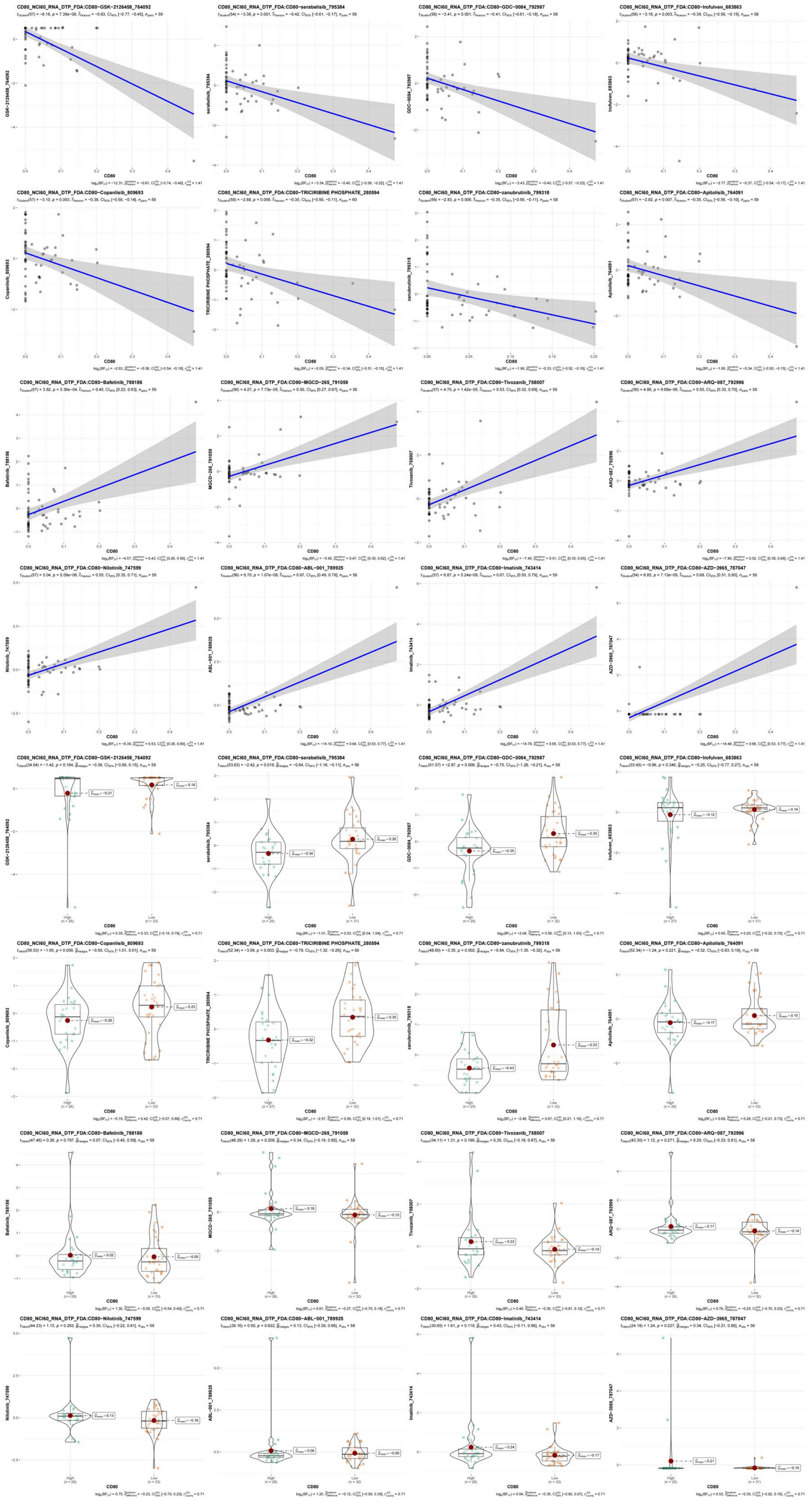

# Supplementary Fig. 4

I

CD86

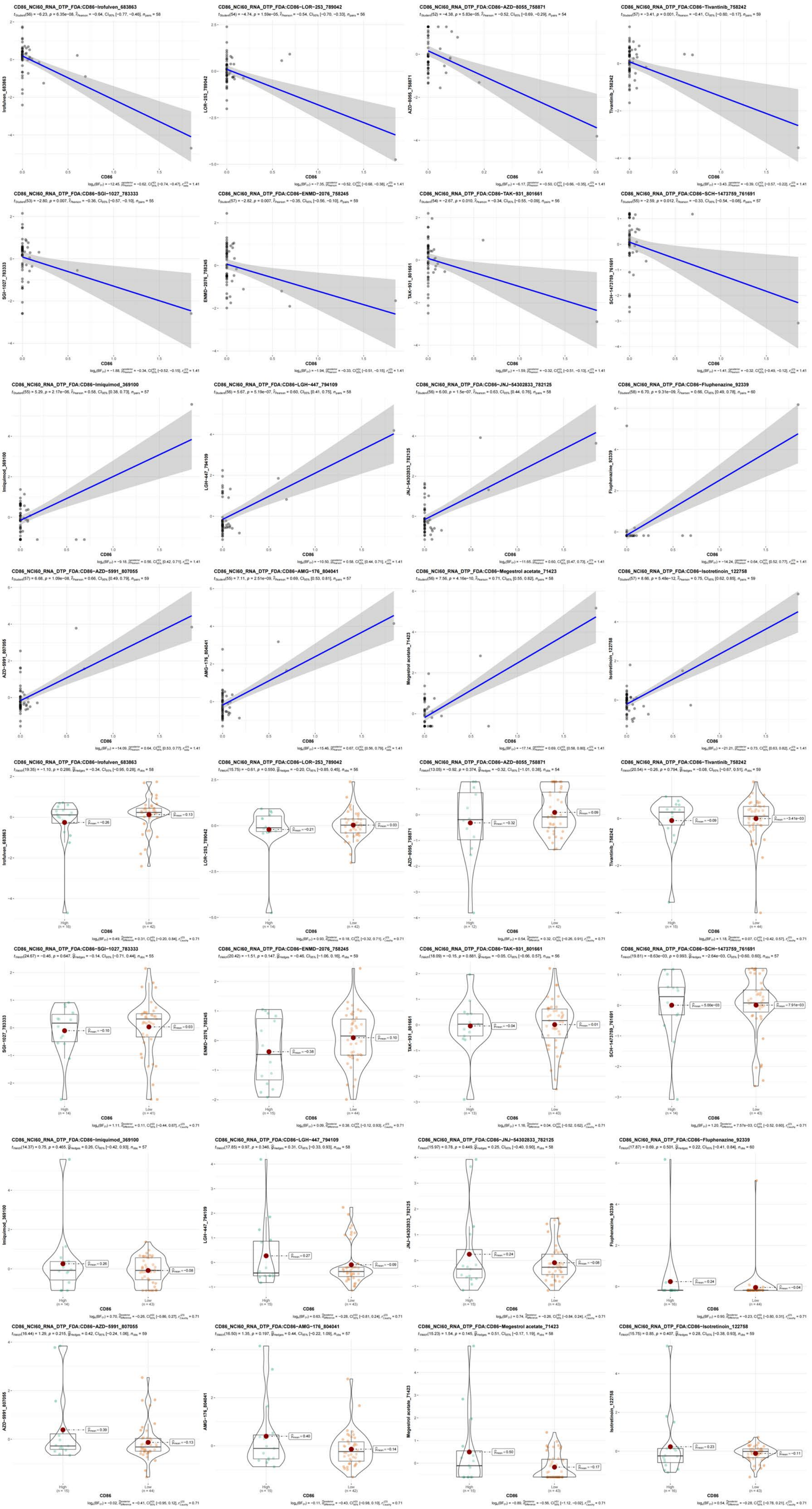

# Supplementary Fig. 4

**J** CD112

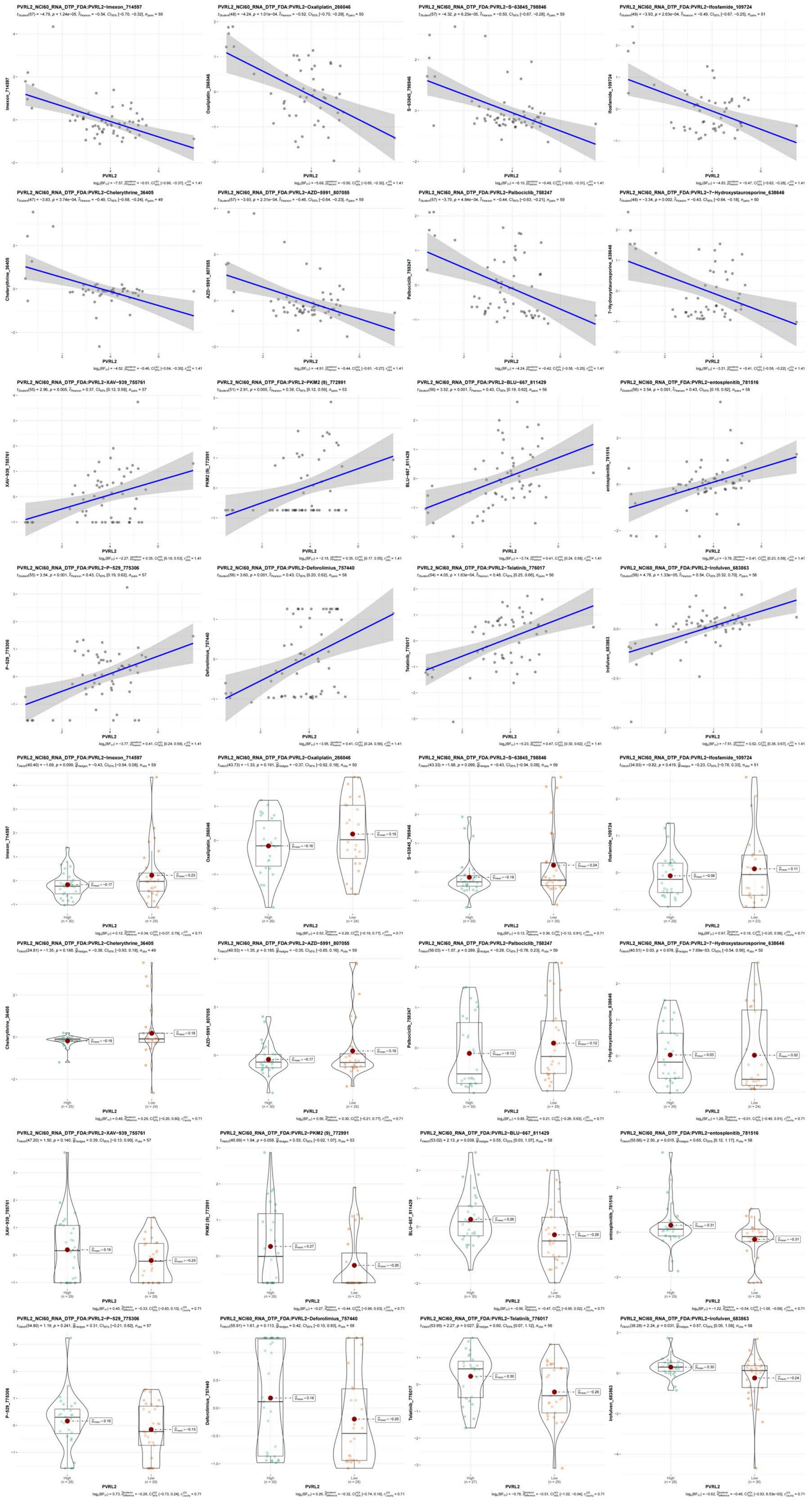

# Supplementary Fig. 4

K CD155

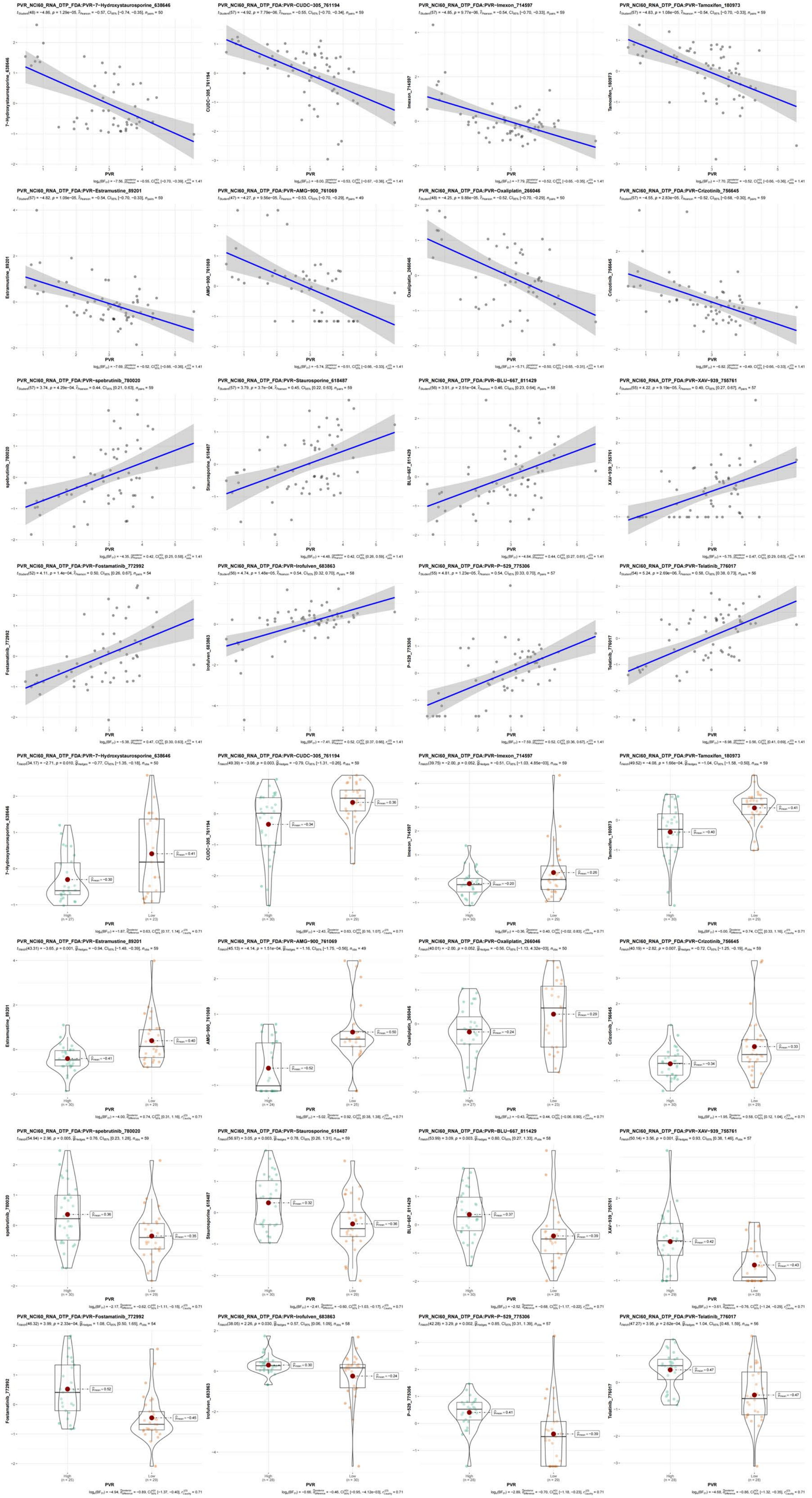

# Supplementary Fig. 4

L FGL1

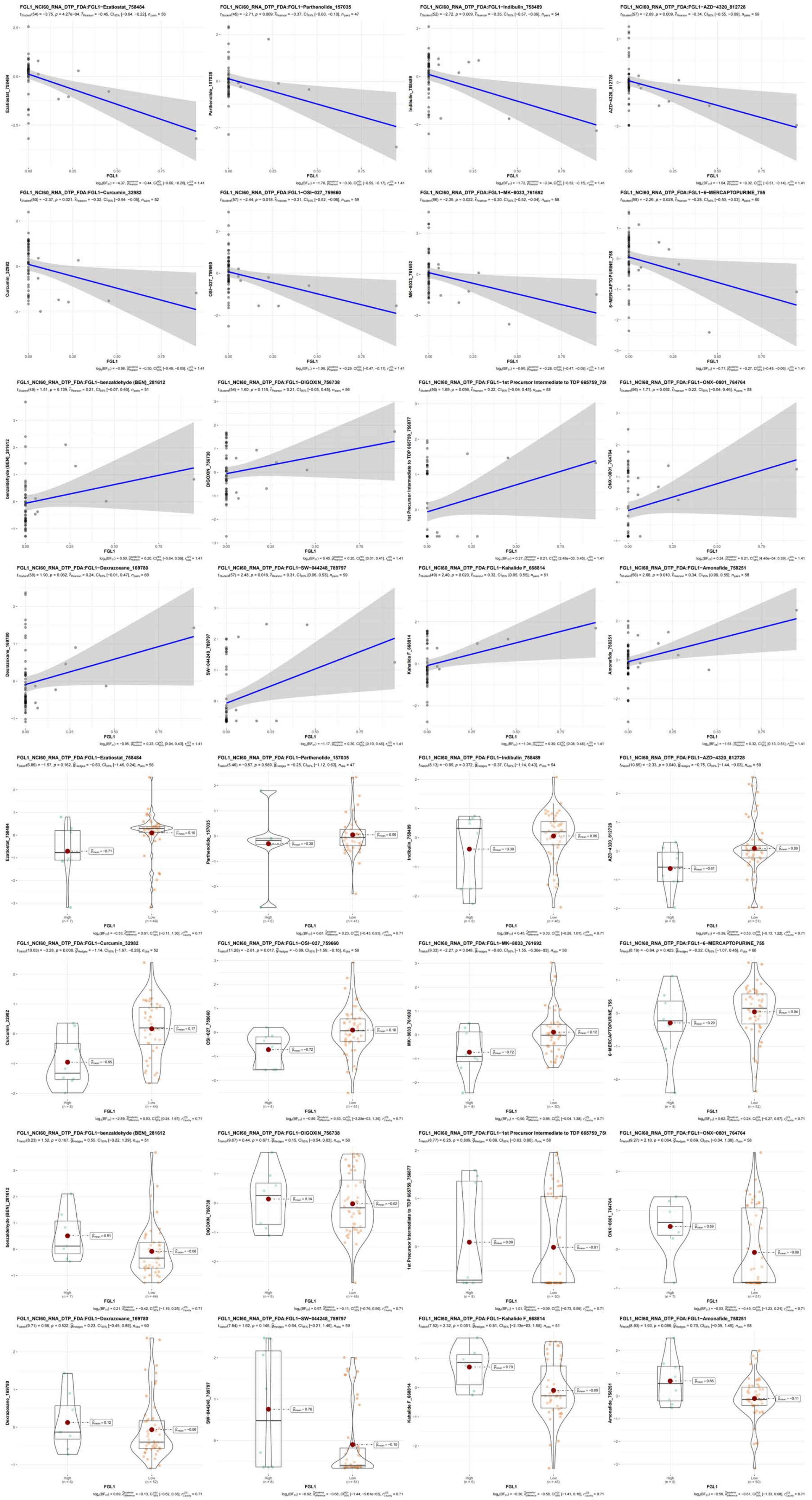

# Supplementary Fig. 4

M HMGB1

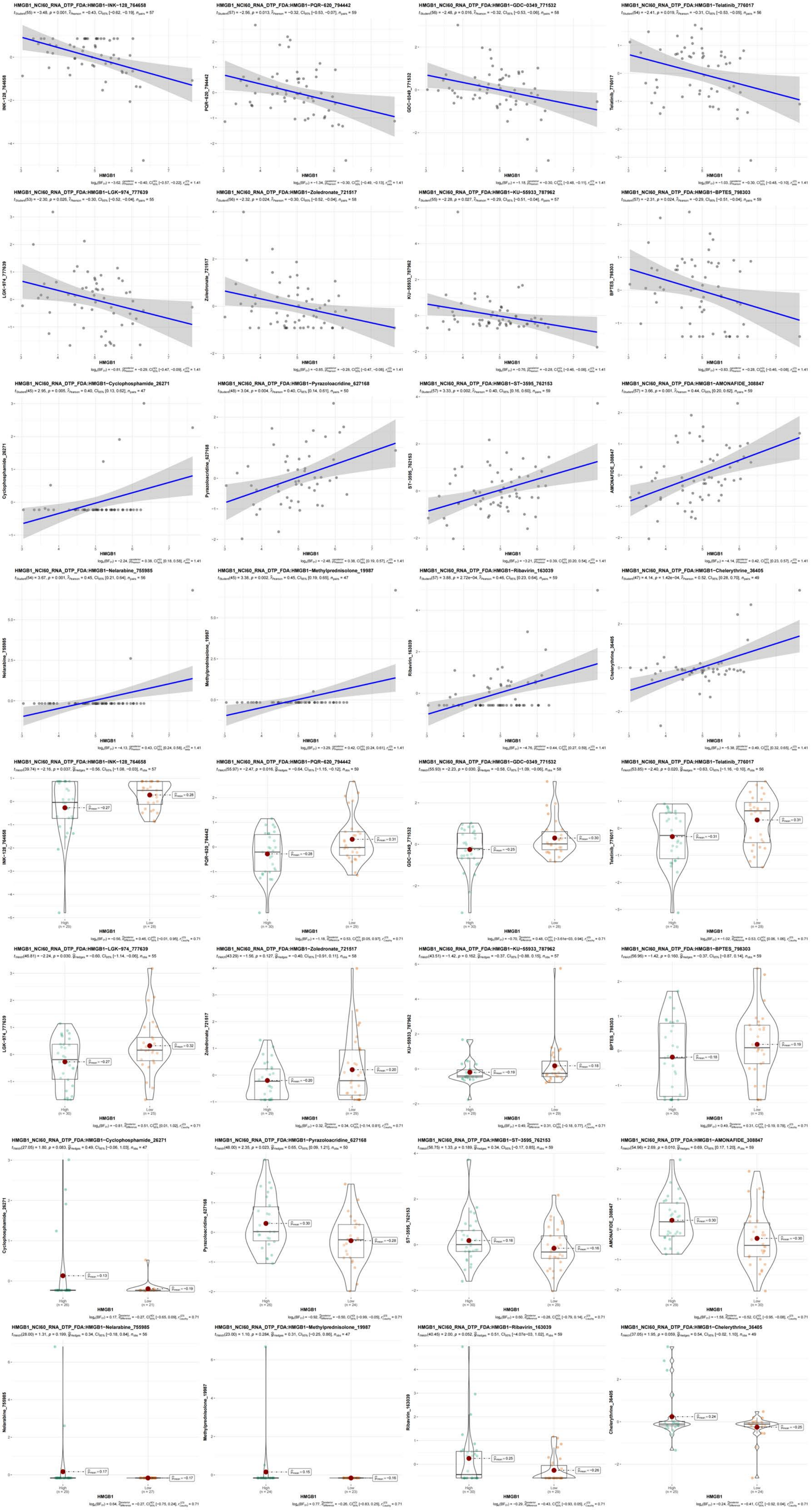

# Supplementary Fig. 4

## N LGALS9

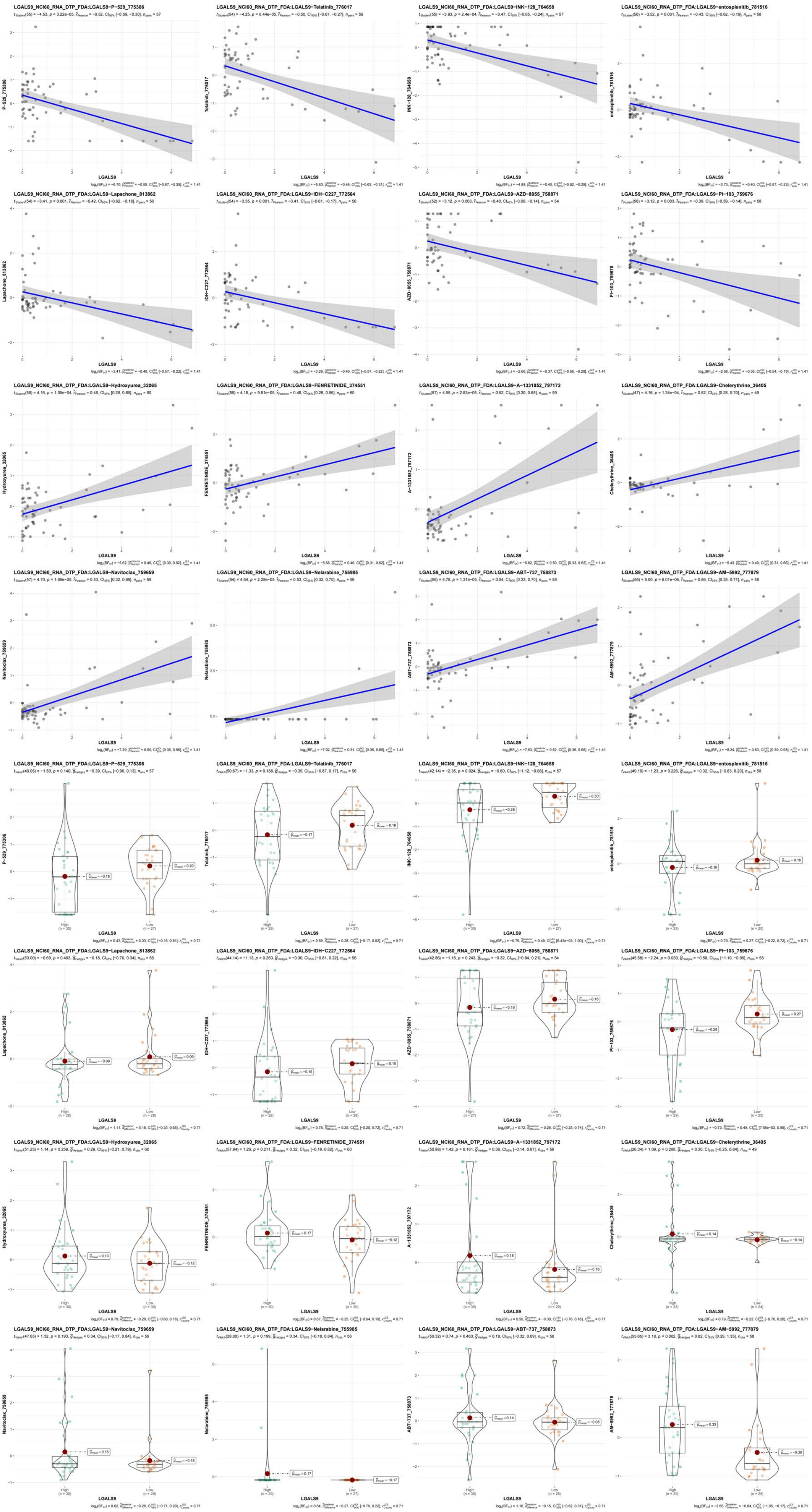

# Supplementary Fig. 4

**O** PD-L1

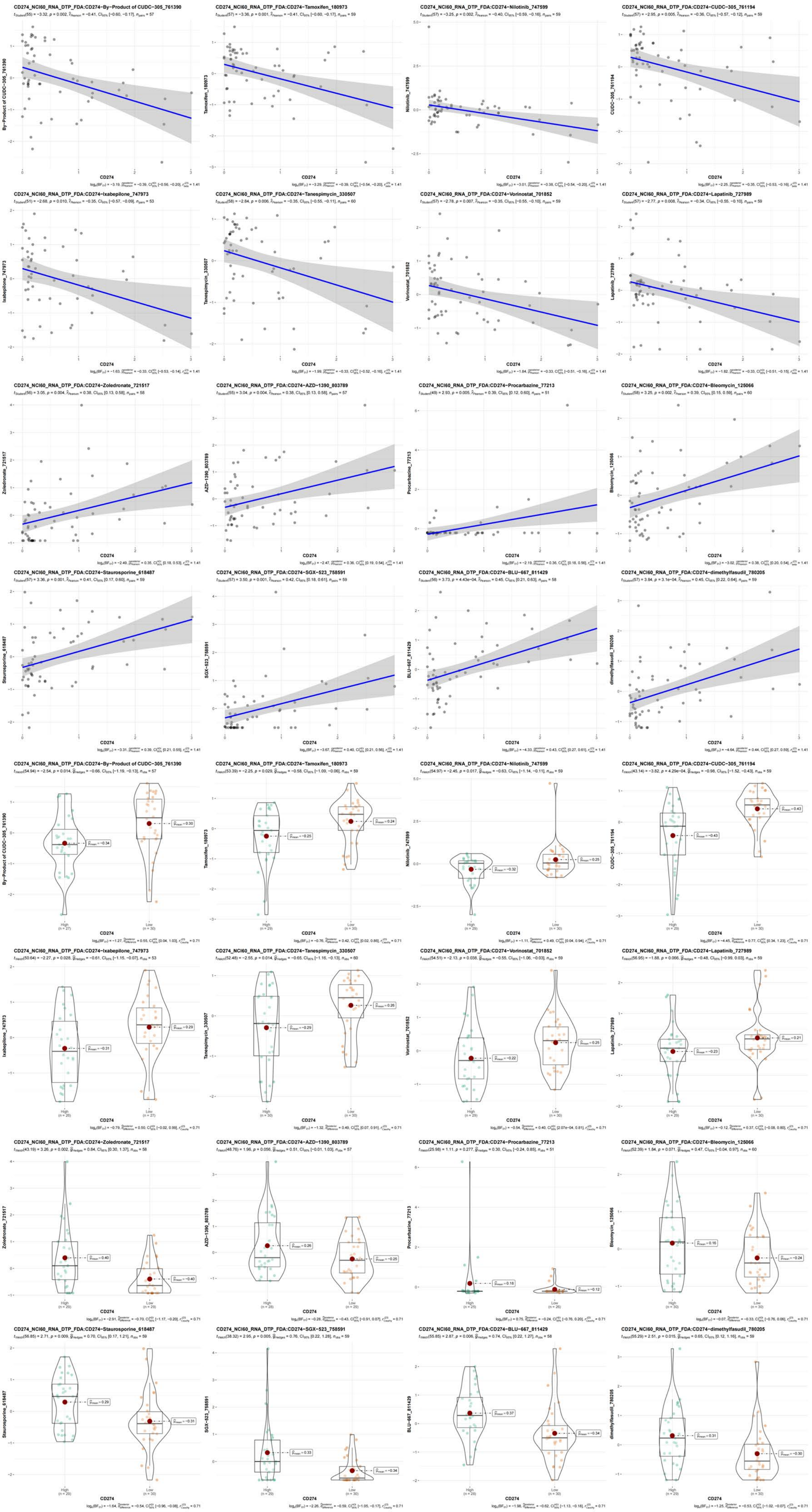

# Supplementary Fig. 4

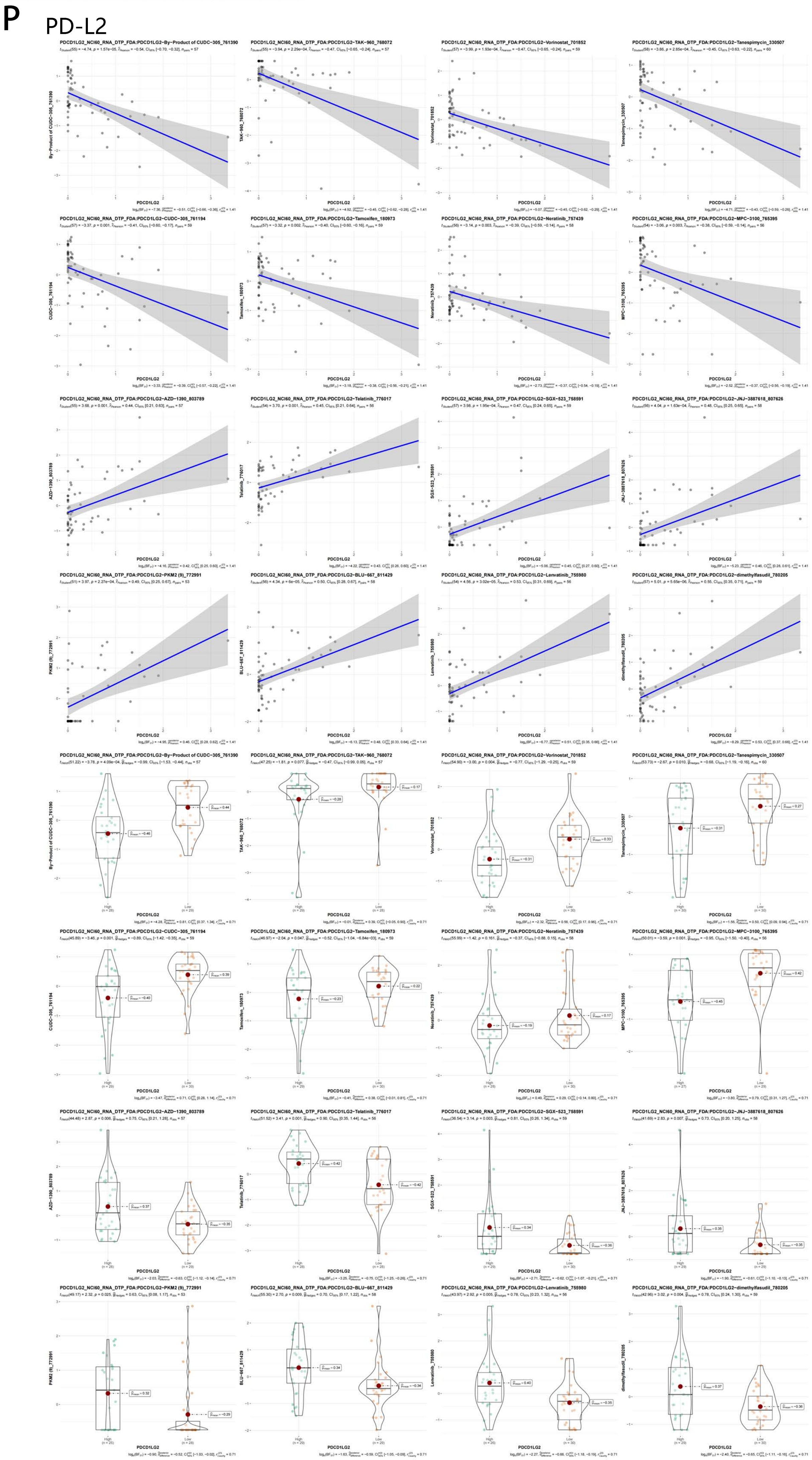

# Supplementary Fig. 4

## Q SEMA4A

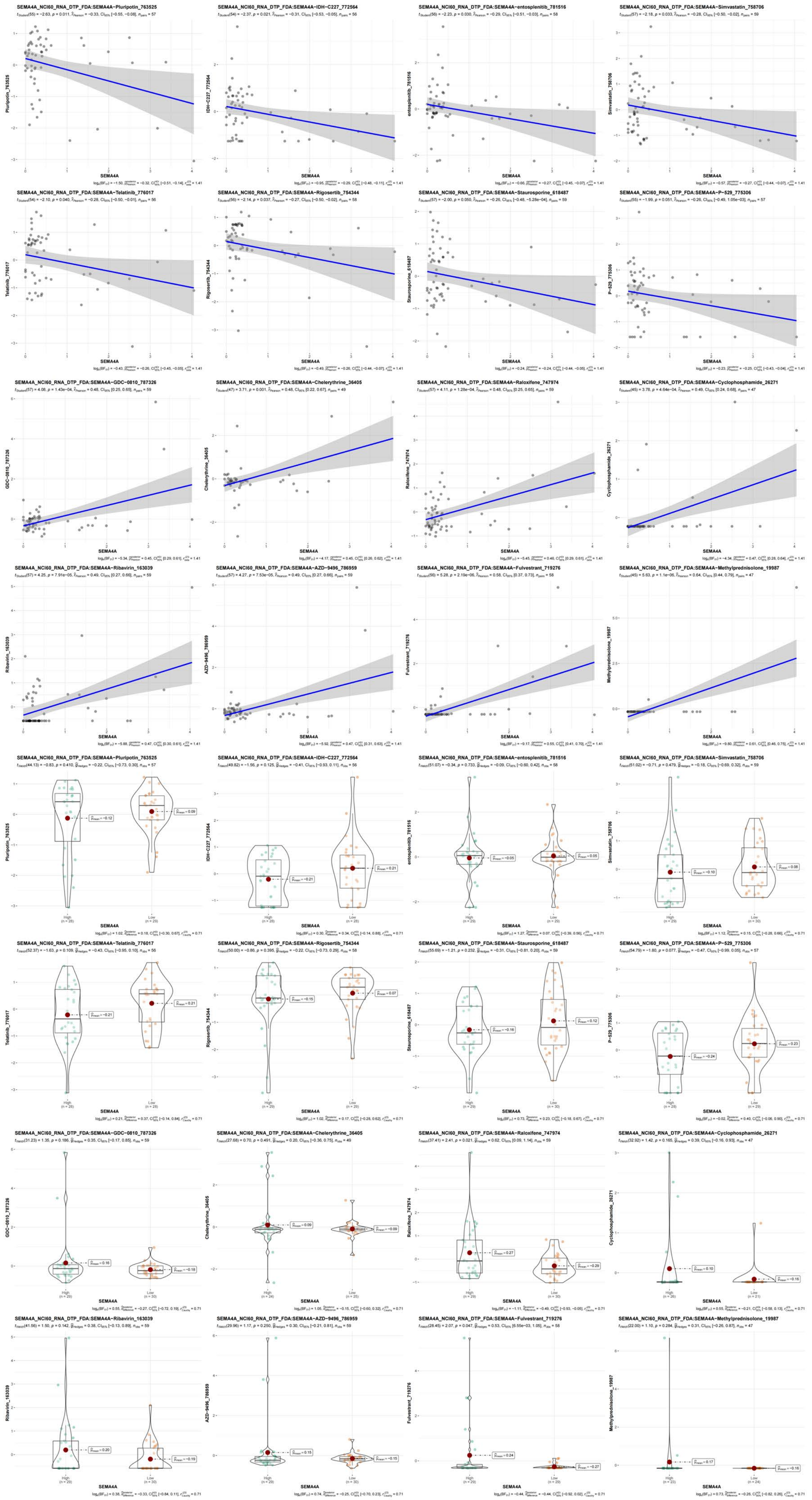

# Supplementary Fig. 4

## R VEGFA

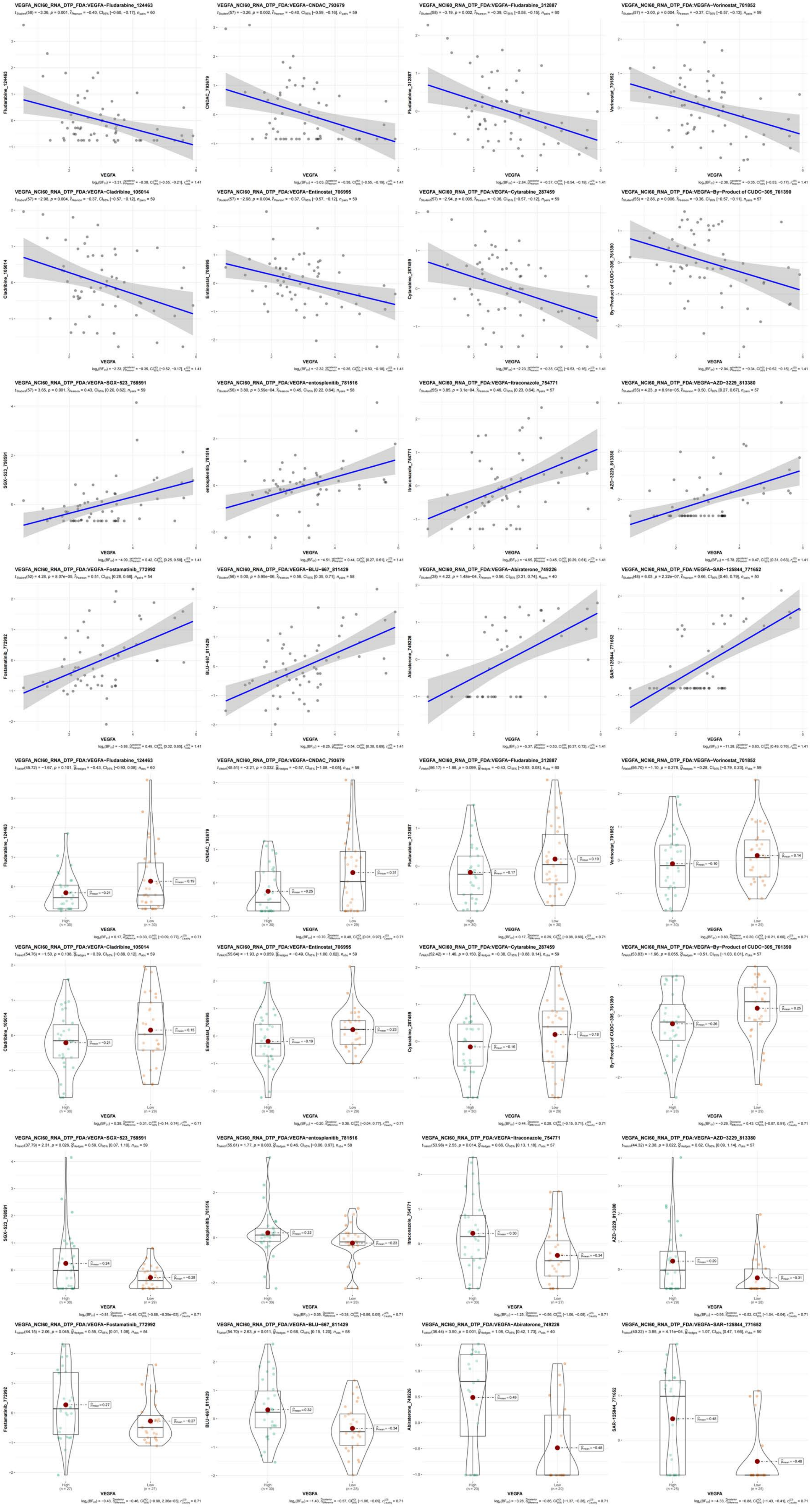

Supplement: Supplementary file 10 [file DataSheet10.pdf]
