# Supplementary material for: Pan-cancer analysis of co-inhibitory molecules revealing their potential prognostic and clinical values in immunotherapy
Source: Front Immunol. 2025 Mar 24;16:1544104. doi: 10.3389/fimmu.2025.1544104 (PMC11973099; doi:10.3389/fimmu.2025.1544104)
Supplement: Supplementary file 11 [file DataSheet11.pdf]

**Figure Legends**

**Supplementary Figure 1.** Expression of Co-inhibitory Molecules and Their Ligands. **(A)** The expression levels of CTLA4, LAG3, NRP1, PD-1, TIGIT, TIM3, VISTA, CD80, CD86, CD112, CD155, FGL1, HMGB1, LGALS9, PD-L1, PD-L2, SEMA4A, and VEGFA in granulocytes, monocytes, T cells, B cells, dendritic cells, NK cells, and progenitor cells. **(B)** The relative expression levels of CD80, CD86, CD112, CD274, PDCD1LG2, FGL1, HMGB1, LGALS9, PVR, PVRL2, SEMA4A, and VEGFA in various cancer types and their corresponding normal tissues.

**Supplementary Figure 2**. Correlation analysis of the expression of co-inhibitory molecules and pan-cancer immune cell infiltration. Data with P-values greater than the threshold ($P > 0.05$, not significant) were cross-labeled. Panels **(A)**-**(E)** represent the correlation between 18 molecules and the infiltration of CD4+ T cells, B cells, neutrophils, macrophages, and myeloid dendritic cells in different tumor types, based on the Spearman method.

**Supplementary Figure 3**. Correlation analysis between VISTA expression and DNA/RNA methylation regulatory genes. **(A)** Circular graph of the relationships between VISTA expression and DNA methylation regulatory genes (DNMT1, DNMT3A, DNMT3B, DNMT3L). **(B)** Circular graph of the relationships between VISTA expression and RNA methylation regulatory genes (ALKBH5, FTO, METTL3, METTL14, WTAP). The peripheral circle

indicated the type of cancer; the different colors of the second circle represented DNA/RNA methylation regulatory genes; the third circle showed the Pearson correlation coefficient; the depth of the fourth ring's color indicated the P-value, and the innermost part was the specific numbers of correlation coefficients and the P-value.

**Supplementary Figure 4**. Analysis of the correlation between co-inhibitory molecules and their ligands with the drug sensitivity of anticancer agents. These drugs include Lxazomib citrate, Vincristine, Tepotinib, MG − 132, Tamoxifen, SB − 590885, Bortezomib, Geldanamycin analog, EGF − 816, Methylprednisolone, BMS − 690514, Sapitinib, Erlotinib, SW − 044248, Dexamethasone, and Gefitinib. The results for **(A)**-**(E)** represent the analysis of CTLA4, LAG3, NRP1, PD-1, TIGIT, TIM3, VISTA, CD80, CD86, CD112, CD155, FGL1, HMGB1, LGALS9, PD-L1, PD-L2, SEMA4A, and VEGFA, respectively.
